# High-Performance Silicon-Rich Microparticle Anodes for Lithium-Ion Batteries Enabled by Internal Stress Mitigation

**DOI:** 10.1007/s40820-023-01190-7

**Published:** 2023-10-09

**Authors:** Yao Gao, Lei Fan, Rui Zhou, Xiaoqiong Du, Zengbao Jiao, Biao Zhang

**Affiliations:** 1 Department of Applied Physics, The Hong Kong Polytechnic University, Kowloon, Hong Kong, People’s Republic of China; 2 Department of Mechanical Engineering, The Hong Kong Polytechnic University, Kowloon, Hong Kong, People’s Republic of China; 3 Department of Physics, The Chinese University of Hong Kong, New Territories, Hong Kong, People’s Republic of China

**Keywords:** Silicon anodes, Silicon microparticles, Lithium-ion batteries, Internal stress

## Abstract

Si is a promising anode material for Li ion batteries because of its high specific capacity, abundant reserve, and low cost. However, its rate performance and cycling stability are poor due to the severe particle pulverization during the lithiation/delithiation process. The high stress induced by the Li concentration gradient and anisotropic deformation is the main reason for the fracture of Si particles. Here we present a new stress mitigation strategy by uniformly distributing small amounts of Sn and Sb in Si micron-sized particles, which reduces the Li concentration gradient and realizes an isotropic lithiation/delithiation process. The Si_8.5_Sn_0.5_Sb microparticles (mean particle size: 8.22 μm) show over 6000-fold and tenfold improvements in electronic conductivity and Li diffusivity than Si particles, respectively. The discharge capacities of the Si_8.5_Sn_0.5_Sb microparticle anode after 100 cycles at 1.0 and 3.0 A g^−1^ are 1.62 and 1.19 Ah g^−1^, respectively, corresponding to a retention rate of 94.2% and 99.6%, respectively, relative to the capacity of the first cycle after activation. Multicomponent microparticle anodes containing Si, Sn, Sb, Ge and Ag prepared using the same method yields an ultra-low capacity decay rate of 0.02% per cycle for 1000 cycles at 1 A g^−1^, corroborating the proposed mechanism. The stress regulation mechanism enabled by the industry-compatible fabrication methods opens up enormous opportunities for low-cost and high-energy–density Li-ion batteries.

**Supplementary Information:**

The online version contains supplementary material available at 10.1007/s40820-023-01190-7.

## Highlights

- The Sn and Sb incorporation boosts the electronic conductivity and lithium diffusivity of Si anodes, thereby reducing the stress due to lithium concentration gradient.
- The lithiation of modified electrode mimics isotropic solid solution reaction, rather than the original anisotropic two-phase reaction of Si, effectively weakening the stress concentration.
- The silicon-rich particles exhibit a capacity of over 1.9 Ah g^−1^ after 100 cycles at 0.1 A g^−1^ and maintain the excellent cyclic stability at 3 A g^−1^.

**Supplementary Information:**

The online version contains supplementary material available at 10.1007/s40820-023-01190-7.

## Graphical abstract

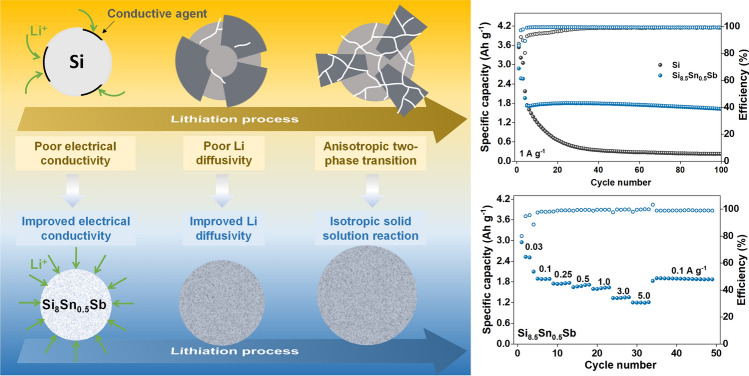

## Introduction

The burgeoning demand for electric vehicles and portable consumer electronics is placing ever-higher requirements on the energy density, charging speed, and lifetime of energy storage devices [1, 2]. Li-ion battery (LIB) is one of the most competitive battery technologies, while its specific capacity is becoming a shortcoming restricting its development [3]. Therefore, Si (3.58 Ah g^−1^), with a specific capacity of almost 10 times that of commercial graphite-based anodes (0.37 Ah g^−1^), becomes an outstanding candidate for LIB anode materials [4–6]. The competitiveness of Si is further enhanced by its appropriate electrochemical potential (~ 0.4 V vs Li/Li^+^) [7], abundant reserve, and environmental benignity. However, Si has several fatal drawbacks as an anode material. One is that compared to intercalation-type graphite anode, the alloy-type Si anode has to withstand a large volume change (~ 300%) during lithiation. The low Li diffusivity of Si results in a large Li concentration gradient and thus high internal stress during cycling, leading to severe particle pulverization and incomplete Li extraction [8–10]. Due to the low conductivity of Si (2.52 $\times$ 10^–6^ S cm^−1^) [11], the pulverized fragments easily become electrical insulated and cannot contribute capacity in subsequent cycles [12–14]. Furthermore, the newly exposed anode surfaces after cracking need to form solid electrolyte interphase (SEI), which consumes a considerable amount of Li ions. Under the combined effect of these factors, the capacity of pure Si anode tends to drop rapidly within a few cycles. The situation gets worse when the particle size of Si anode increases [15, 16].

The volume change during lithiation/delithiation is found not to cause electrode fracture when the Si particle size is less than 150 nm [10, 15]. Nanotechnology has consequently become the most common solution to improve the stability of Si anodes. Traditional methods include the use of Si nanoparticles [17], nanotubes [18], nanowires [19–21], thin films with nanoscale thickness [22], and rational structural design [23], such as nanoporous Si [24] and hollow shell structures [25, 26] that retain the volume expansion space. However, nanoengineered Si anodes suffer from some intractable defects. First, the low tap density of nanostructured Si potentially reduces the volumetric energy density. Secondly, the low conductivity of Si requires compounding with other conductive materials to build a three-dimensional conductive network, which further reduces the energy density of battery. Moreover, the large surface area of nanomaterials consumes a large Li^+^ inventory in the formation of SEI. Finally, the complex and delicate fabrication process of nano-Si anodes greatly weakens the low-cost advantage of Si. These thorny flaws of nano-Si anodes have made micron-sized Si anode an increasingly important research direction.

For micron-sized Si anodes, particle fracture during cycling is one of the most critical issues to be addressed. Preparation of robust protective layers in forms of binder [27–31], coating [32–36], or SEI [37] is currently the predominant approach, but its effectiveness decreases with increasing particle size. In such cases, eliminating or managing the stressor causing particle fracture could be a promising solution. The internal stress of Si particles generated during cycling mainly originates from the Li concentration gradient and the phase-transition process involving anisotropic deformation [15, 16]. Therefore, an effective mechanism needs to cover two aspects. One is to increase the Li diffusivity to reduce the Li concentration gradient within the anode particles, and the other is to make the lithiation/delithiation process of the anode to be isotropic, similar as that of amorphous Si.

Doping or alloying Si with other elements are promising approaches to improve its electron/Li conductivity [38]. Although B-doped Si micro-rod anode has been reported to exhibit improved cycling performance [39], there are few reports on the modification of Si micron-sized anodes by alloying with other elements. We focus on Sn (electrical conductivity: 9.17 $\times$ 10^4^ S cm^−1^) and Sb (electrical conductivity: 2.88 $\times$ 10^4^ S cm^−1^) [11] with a high Li theoretical capacity, high electron/Li conductivity, and abundant reserves to modify Si without sacrificing its low-cost and high-capacity advantages [40–45]. So far, the reported methods of modifying Si anodes with Sn and Sb are mainly focused on nanomaterials, such as to form Si–Sn composite electrode sheets by co-sputtering [46] or hot-pressing [47], to prepare Sn ribbon entangled Si particles by mechanical milling [48], to synthesize Sn-seeded Si nanowires [49] or Sn nanoparticle-embedded mesoporous-Si [50], and to form nanoporous Si–Sb anodes via dealloying [51, 52]. These approaches have yielded significant improvements in battery cycling performance, but are difficult to scale up to micron-sized Si anodes. To eliminate stress concentrations during electrode reactions, it is ideal to form an amorphous single-phase alloy with Si, Sn, and Sb. However, the high melting temperature of Si (1414 °C) and the ultra-low solid solubility of Sn and Sb in Si at thermodynamic equilibrium state prevent conventional alloy preparation techniques from achieving this goal.

In this work, we propose a stress mitigation strategy to realize the stable cycling of Si-rich microparticle anodes via a simple two-step fabrication method. Bulk Si, Sn, Sb raw materials were first smelted into Si_8.5_Sn_0.5_Sb alloy ingots through an electric arc furnace. High-energy ball milling was then used to pulverize the ingot and further promote the mixing of the three elements. The Si_8.5_Sn_0.5_Sb microparticle anode demonstrated tremendous improvements in electron/Li conductivity, mechanical integrity, and consequently the cycling performance compared to Si anode. After 100 cycles in conventional carbonate-based electrolyte, the Si_8.5_Sn_0.5_Sb microparticle anode exhibited discharge capacities of 1.62 and 1.19 Ah g^−1^ for the rate of 1 and 3 A g^−1^, respectively, and the capacity retention were both higher than 90%. The stress mitigation mechanism was substantiated by finite element simulation results and extended to quaternary and pentanary alloy systems, both of which yielded extraordinary cyclic stability.

## Experimental Section

### Preparation of Anode Materials and Electrodes

Alloy ingots with a nominal composition of Si_8.5_Sn_0.5_Sb (at%) were prepared by arc-melting a mixture of raw Si, Sn, and Sb (purity > 99.9 wt%) under a Ti-gettered argon atmosphere. The melting was repeated at least four times to ensure chemical homogeneity. The alloy ingot was pre-crushed into half-centimeter-sized pieces and sealed into a 100-mL ball-milling jar under an argon atmosphere. The mass ratio of ball to alloy sample was approximately 20:1. The sample was milled (SPEX 8000) eight times for 30 min each at 15 min intervals. The ball-milled particles were sieved using a 300-mesh sieve and stored in a dry argon atmosphere. The arc-melted and ball-milled alloy particles were mixed with super P by ball milling for half an hour, and then mixed with vapor-grown carbon fibers and sodium carboxymethyl cellulose in deionized water for twelve hours. The mass ratio in sequence was 6.4:1.6:1:1. The mixture slurry was cast on a Cu foil (1.2 cm in diameter) and dried in a vacuum oven at 80 °C for 24 h. The mass loading of active materials per anode sheet was 0.75 ± 0.16 mg cm^−2^.

### Half Cells for Electrochemical Testing

Half-cells were assembled with CR2032 coin cells in an argon-filled glove box (H_2_O < 0.5 ppm, O_2_ < 0.5 ppm) using a piece of glass fiber (Whatman, GF/D) separator and Li foil as the counter electrode. The electrolyte was 1 M LiPF_6_ dissolved in ethylene carbonate (EC, DoDochem, purity 99.95%)-dimethyl carbonate (DMC, DoDochem, purity 99.8%) solution at the volume ratio of 1:1, with 5 vol% of fluoroethylene carbonate (FEC, Sigma-Aldrich, 99% pure) as additive. Each coin cell contained 80 µL of electrolytes and was tested at 293 K. Electrochemical impedance spectroscopy (EIS) test was performed on an electrochemical workstation (Biologic SP150) between 100 kHz and 0.1 Hz with an amplitude of 5 mV. Cyclic voltammetry (CV) data were collected on an electrochemical workstation (Solartron Analytical 1400) at 0.05 mV S^−1^ between 0 and 1.5 V. Galvanostatic intermittent titration technique (GITT) tests were collected on an Arbin battery system at 0.1 A g^−1^ with a current pulse of 0.25 h and a relaxation time of 3 h. The batteries underwent 10 cycles at 1 A g^−1^ before the GITT test.

### Material Characterization

The arc-melted alloy ingot was mechanically polished to 0.05 μm before examining its microstructure by using scanning electron microscopy (SEM, Tescan VEGA3). Particle samples for SEM measurements were prepared by sticking the alloy particles on the conductive glue of the sample stage with a toothpick. The size and element distribution of anode particles were evaluated by SEM. The transmission electron microscope (TEM, JEOL JEM-2100F) samples were prepared by dropping the particle-alcohol solution on a copper grid. The elemental distribution and microstructure within individual particles were characterized by energy-dispersive X-ray spectroscopy (EDS) and high-resolution transmission electron microscope (HRTEM) images. To test the electrical conductivity of prepared anode particles, they were first formed into pallets of 1 cm diameter under an isostatic pressure of 250 MPa. The thickness of the pallet was obtained by the vernier caliper. The pallet was then sandwiched between two stainless steel plates and sealed in a coin cell. Resistance was tested by EIS with amplitudes of 100 and 500 mV. The formula for calculating the electronical conductivity is *k* = *d*/(RA), where d and A is the thickness and cross area of the pallet, respectively, and R is the measured resistance. The influence of porosity in the pallet on the measured resistance was ignored here. In-situ XRD test was performed using a Swagelok cell equipped with a beryllium window. In-situ atomic force microscope (AFM, Bruker Icon) testing was performed in an argon-filled glove box using an instrument-equipped electrochemical cell to characterize the evolution of electrode morphology during cycling. For ex-situ characterizations (e.g., SEM, TEM, XRD and AFM) of the electrode after cycling, the cells were disassembled and the electrodes were rinsed with DMC in an argon-filled glove box to remove the electrolyte. For ex-situ TEM samples, the electrode material was scraped from the Cu foil and dispersed in DMC. A drop of the suspension was then deposited on the copper grid and dried in the argon-filled glove box. For ex-situ XRD samples, the electrode sheets were sealed in the homemade in-situ XRD cell in the argon-filled glove box before testing.

## Results and Discussion

### Design Principle and Structural Characterizations

**Fig. 1:**
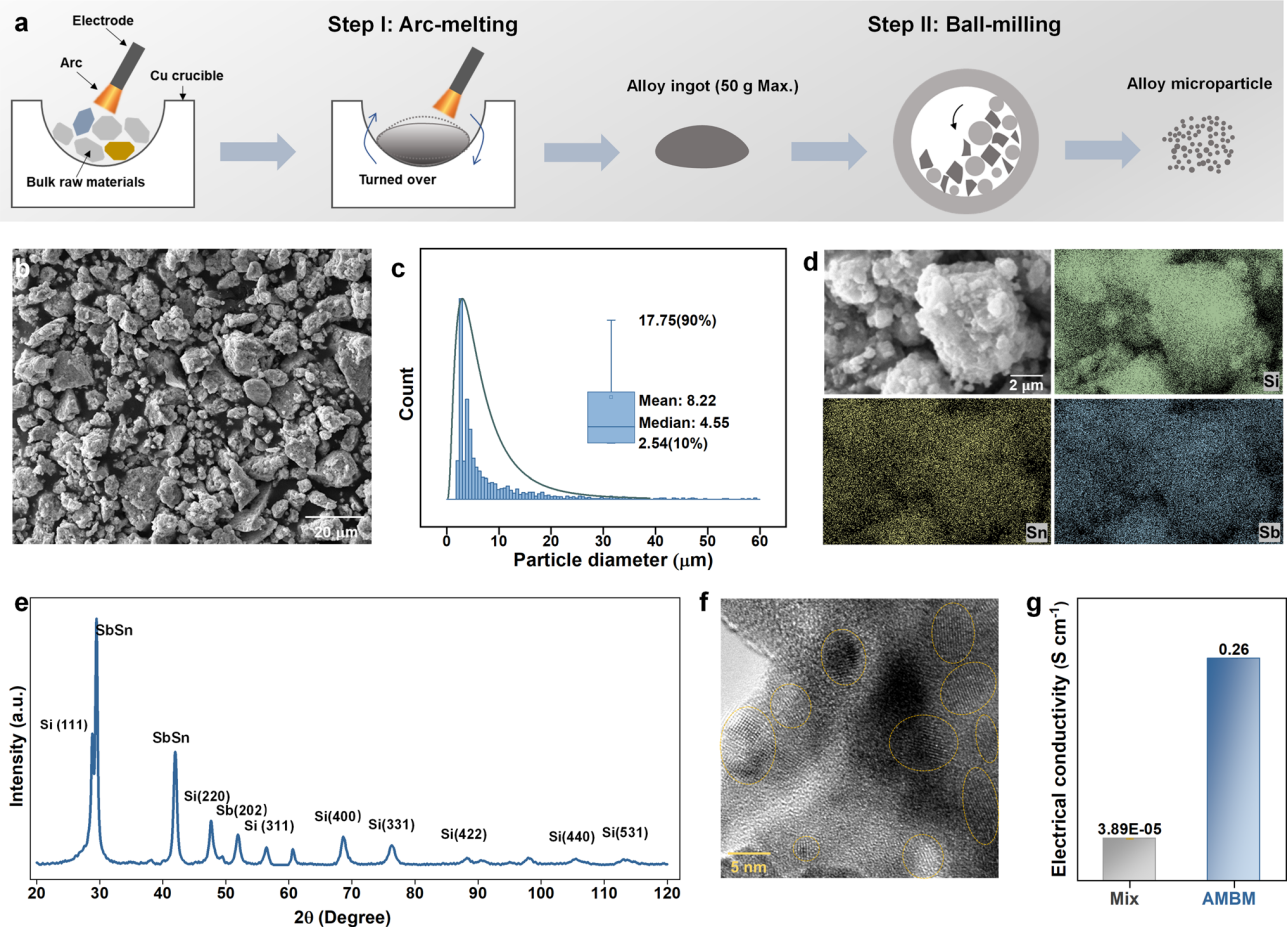
Conceptual design, fabricating process and microstructure. **a** Illustration of combining arc-melting and high energy ball milling to prepare concentration modulated Si-rich (Si_8.5_Sn_0.5_Sb) microparticles. **b** SEM image, **c** granularity statistics, **d** EDS mapping, **e** XRD spectrum, and **f** HRTEM image of prepared microparticles. **g** The measured electrical conductivity of Si_8.5_Sn_0.5_Sb-Mix and Si_8.5_Sn_0.5_Sb-AMBM particles

The stress mitigation mechanism to stabilize Si-rich microparticle anode was achieved by combining arc melting and high-energy ball milling to uniformly distribute Sn and Sb in Si (Fig. 1a). Bulk raw materials were first arc-melted into an alloy ingot (~ 45 g, Fig. S1), and the SEM and EDS results (Fig. S2) show a multiphase interwoven structure. The alloy ingot was then ball-milled into particles with an average particle size of 8.22 μm (Fig. 1b, c). EDS and XRD tests were performed on the alloy particles obtained after high-energy ball milling. Small peaks corresponding to the Sb and SnSb phases can still be found in the XRD spectrum (Fig. 1e), but no obvious phase boundaries were observed in the EDS image (Fig. 1d). This indicates that although no single-phase alloy is obtained, high-energy ball milling can greatly promote the homogeneous mixing of Si, Sn, and Sb. After high-energy ball milling, the grain size of the matrix Si phase is also considerably reduced. Fitting the half-peak width of Si in the XRD pattern using the Williamson–Hall method [53] yields an average Si grain size of approximately 38 nm (Fig. S3); this structural feature was also visually observed in the HRTEM image (Figs. 1f and S4).

Due to the high toughness and ductility of Sn, if commercial Si, Sn, and Sb powders are directly mixed by high-energy ball milling, the so-called “cold welding” effect can lead to the self-agglomeration and growth of Sn powders [48]. Pre-forming the alloy by arc melting can effectively avoid the cold-welding effect in the subsequent ball milling step and achieves uniform mixing of these elements. The molar ratio of Si, Sn and Sb before arc melting was 8:1:1. Due to the low melting point of Sn and Sb, there were some losses during the smelting process. Finally, based on multiple EDS data statistics, the molar ratio of Si, Sn and Sb in the final prepared alloy microparticles was estimated to be 8.5:0.5:1. For convenience, this sample is referred to as Si_8.5_Sn_0.5_Sb-AMBM, where AMBM is short for “arc melting—ball milling”. As shown in Fig. 1g, the Si_8.5_Sn_0.5_Sb-AMBM microparticles has an over 6000-fold increase in electrical conductivity compared to Si powders.

### Electrochemical Performance

**Fig. 2:**
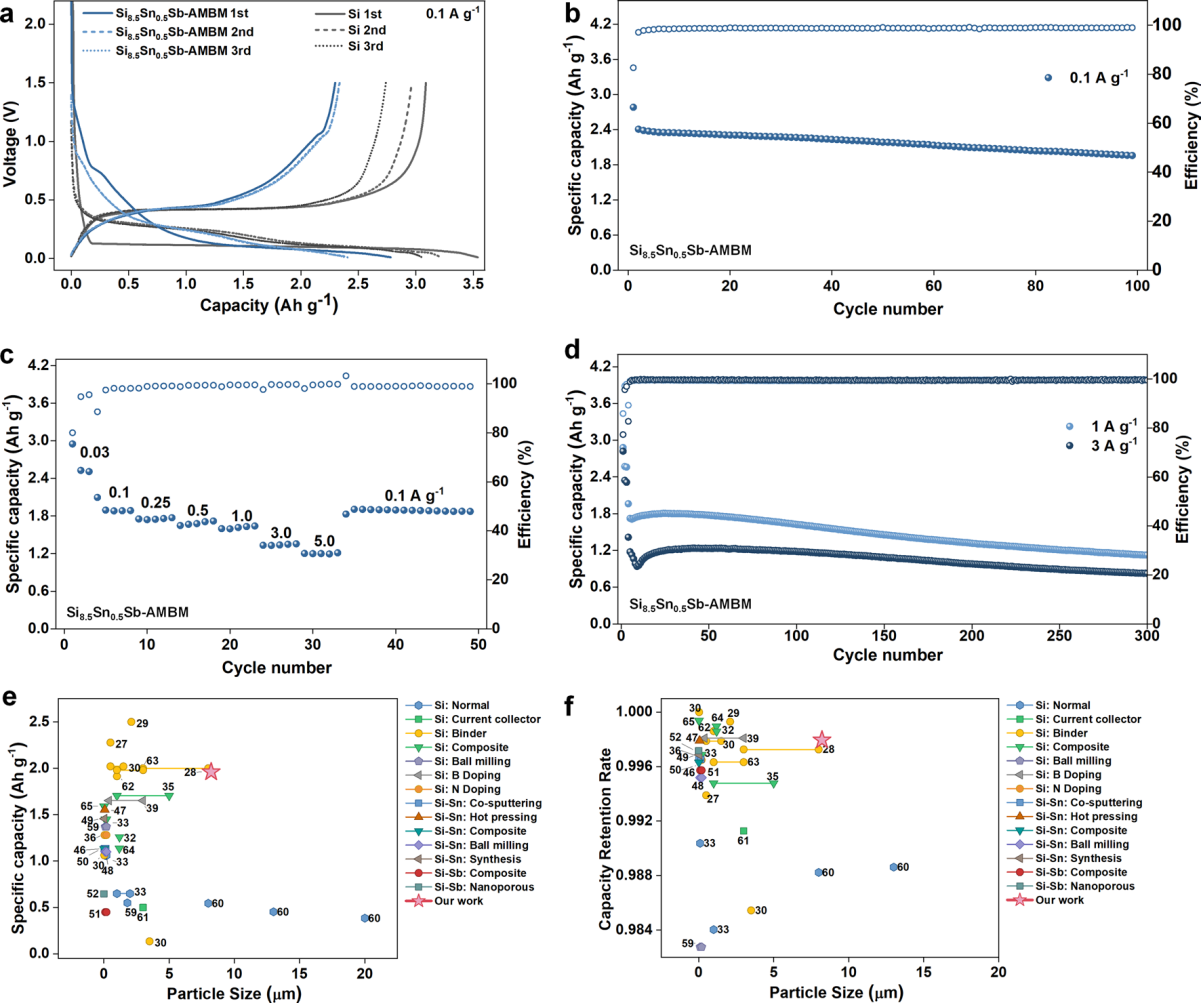
Electrochemical performance. **a** Galvanostatic curves of the first three cycle for Si and Si_8.5_Sn_0.5_Sb-AMBM anodes at 0.1 A g^−1^. **b** Cycling performance of Si_8.5_Sn_0.5_Sb-AMBM anode at 0.1 A g^−1^. **c** Discharge capacity and CE of Si_8.5_Sn_0.5_Sb-AMBM anode obtained at different current densities. **d** Cycling performance of Si_8.5_Sn_0.5_Sb-AMBM anode at 1 and 3 A g^−1^. The summary of **e** the relationship between particle size and reversible capacity, and **f** the relationship between particle size and capacity retention rate per cycle, for the reported pure Si microparticle anodes and Si anodes modified with Sn or Sb

The performance of Si_8.5_Sn_0.5_Sb-AMBM microparticles as LIB anode was evaluated in coin cell tests in a conventional carbonate-based electrolyte (Fig. 2). A mixture of commercial Si, Sn, and Sb powders (referred to as Si_8.5_Sn_0.5_Sb-Mix, EDS results in Fig. S5) were used to prepare the control group anode. Silicon anodes are usually cycled within a voltage window narrower than its theoretical one to avoid the severe pulverization [37, 54]. We also took this protocol when the battery was cycled at current densities larger than 0.1 A g^−1^. Specifically, cells were cycled between 0.01 and 1.5 V at 0.1 A g^−1^. For other conditions, cells were first pre-cycled 3 times between 0.01 and 1.5 V at small current densities, and then cycled between 0.06 and 1 V at the targeted current densities.

Voltage profiles of the initial three cycles for Si_8.5_Sn_0.5_Sb-AMBM and control group anodes tested at 0.1 A g^−1^ between 0.01 and 1.5 V (vs. Li/Li^+^) are shown in Figs. 2a and S6, respectively. During the initial lithiation of pure Si anode, a broad and flat voltage plateau at ~ 0.1 V was observed, while during the subsequent cycling, the voltage plateau disappears and the discharge curve changes to a sloped shape [55]. The voltage curve of the Si_8.5_Sn_0.5_Sb-Mix anode exhibits a staircase shape, corresponding to multiple phase transition voltage plateaus for the three elements. The Si_8.5_Sn_0.5_Sb-AMBM anode presents completely different voltage curves compared with the above two samples. A short charging plateau corresponding to the lithiation of Sb appears at ~ 0.85 V [56, 57] in the first discharge cycle and disappears in following cycles. Then from 0.75 to 0.01 V, the first cycle voltage curve exhibits a slope with no apparent plateaus and the curve starts to slop from 0.9 V in the second cycle, which is far earlier than the value of 0.45 V in the case of pure Si anode. The sloping voltage curve of Si_8.5_Sn_0.5_Sb-AMBM anode indicates that its lithiation/delithiation process is closer to a solid solution reaction than a two-phase reaction [58]. At a current density of 0.1 A g^−1^, the first discharge and charge capacities of the Si_8.5_Sn_0.5_Sb-AMBM anode are 2.78 and 2.30 Ah g^−1^, respectively, corresponding to the initial Coulombic efficiencies (CE) of 82.62%. The irreversible capacity may be caused by electrolyte decomposition to form SEI and incomplete delithiation. As shown in Fig. 2b, the specific discharge capacity of the Si_8.5_Sn_0.5_Sb-AMBM anode battery after 100 cycles remains 1.96 Ah g^−1^. Under the same test condition, the Si_8.5_Sn_0.5_Sb-Mix anode of the comparison group only retains a discharge capacity of 0.21 Ah g^−1^ (Fig. S7). The cycling performance of the Si_8.5_Sn_0.5_Sb-AMBM anode is also superior when the mass loading is high. As shown in Fig. S8, a discharge capacity of 1.14 Ah g^−1^ is obtained after 100 cycles’ cycling at 0.5 A g^−1^ with a mass loading of 1.60 mg cm^−2^. 

Figure 2c presents the performance of Si_8.5_Sn_0.5_Sb-AMBM anode at seven different current densities. The battery was first activated at 0.03 A g^−1^ for 3 cycles and then tested for 5 cycles at each subsequent rate [37, 54]. The voltage curves of Si_8.5_Sn_0.5_Sb-AMBM anode at different current densities all exhibit a slop shape (Fig. S9), indicating that the battery process is dominated by solid-solution reactions. The discharge capacities are 1.85, 1.73, 1.67, 1.61, 1.34 and 1.19 Ah g^−1^ for rates of 0.1, 0.25, 0.5, 1.0, 3.0 and 5.0 A g^−1^, respectively. After cycling at 5.0 A g^−1^, the battery is again cycled at 0.1 A g^−1^, recovering almost 100% of its capacity. These results indicate that the Si_8.5_Sn_0.5_Sb-AMBM anode has superior rate performance and cycling stability. Long-cycle performance of Si_8.5_Sn_0.5_Sb-AMBM and comparison group anodes at 1 and 3 A g^−1^ are presented in Figs. 2d and S10, respectively. The decrease in capacity for the first 10 cycles at 3 A g^−1^ may be due to the fact that at high current density, the relative large anode particles are more prone to breakage during the initial several cycles and generate new electrode surface that triggers the continuous SEI formation. The discharge capacities of Si and Si_8.5_Sn_0.5_Sb-Mix anodes (Fig. S10) drop rapidly to 0.23 and 0.24 Ah g^−1^, respectively, after 100 cycles at 1 A g^−1^, corresponding to a 13% and 14% capacity retention with respect to the first discharge capacity after pre-cycling, respectively. In contrast, the Si_8.5_Sn_0.5_Sb-AMBM anode shows substantially improved cycling stability, whose discharge capacity is 1.62 (94.2% capacity retention) and 1.28 Ah g^−1^ (99.6% capacity retention) after 100 cycles at 1 and 3 A g^−1^, respectively. Furthermore, the reversible capacities of Si_8.5_Sn_0.5_Sb-AMBM anode after 300 cycles at 1 and 3 A g^−1^ remain at the values of 1.13 and 0.82 Ah g^−1^, respectively, corresponding to the capacity retention rates for each cycle of 99.86% and 99.88%, respectively.

We summarize in Fig. 2e, f the relationships between the particle size, the reversible capacity after 100 cycles (50 cycles for Refs [46, 48, 59].), and the capacity retention rate per cycle for the reported Si microparticle anodes and nanoengineered-Si anodes modified with Sn or Sb [27–30, 32, 33, 35, 36, 39, 46–52, 59–65]. The mass loading of active materials in electrodes shown in Fig. 2e, f are listed in Table S1. The Si_8.5_Sn_0.5_Sb-AMBM anode performs particularly well in achieving high capacity and stable cycling over large-particle Si anodes.

The charge transfer resistances of anodes were assessed by EIS measurements. Figure S11a shows the Nyquist plots measured at open circuit potential for Si_8.5_Sn_0.5_Sb-AMBM and Si_8.5_Sn_0.5_Sb-Mix anodes after 3 cycles. The results of the charge transfer resistance (*R*_ct_) obtained after fitting the equivalent circuit [66] are summarized in Fig. S11b. The battery with Si_8.5_Sn_0.5_Sb-AMBM anode has a much smaller *R*_ct_ (18.9 $\Omega$) than the Si_8.5_Sn_0.5_Sb-Mix anode (424.6 $\Omega$). This indicates that the Si_8.5_Sn_0.5_Sb-AMBM anode has better reactivity and faster charge transport kinetics. GITT tests were performed to evaluate the diffusivity of Li in anode particles (Fig. S12) [47, 67]. For most voltage ranges, the Li diffusion coefficient of the Si_8.5_Sn_0.5_Sb-AMBM anode is over 10 times higher than that of the Si_8.5_Sn_0.5_Sb-Mix anode (Fig. S13). The EIS and GITT results imply that our proposed fabrication method can effectively enhance the electron/Li conductivity of Si microparticle anodes. The Sn and Sb network in Si provides pathways for electron and Li diffusion throughout the electrode surface and inside.

### Electrode Reaction Mechanism

**Fig. 3:**
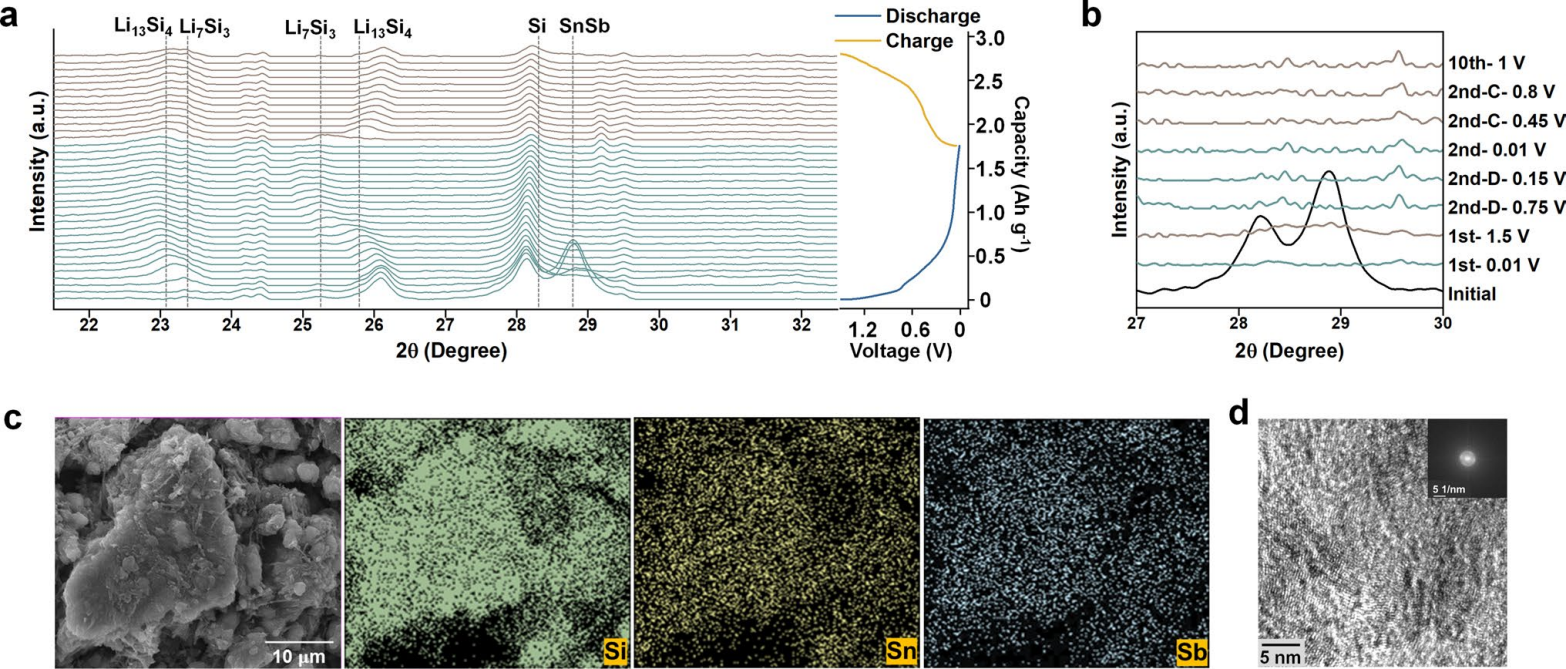
Electrode reaction mechanism. **a** In-situ XRD results of Si_8.5_Sn_0.5_Sb-AMBM anode for the first discharge/charge cycle. **b** Ex-situ XRD results of Si_8.5_Sn_0.5_Sb-AMBM anode. **c** EDS mapping and **d** HRTEM of Si_8.5_Sn_0.5_Sb-AMBM anode after 3 cycles at 0.1 A g^−1^ from 0.01 to 1.5 V

In-situ XRD measurements were carried out on the first cycle of Si_8.5_Sn_0.5_Sb-AMBM (Fig. 3a) and Si_8.5_Sn_0.5_Sb-Mix (Fig. S14) anodes to investigate the mechanisms of electrode reactions. Figure 3a shows the XRD pattern with the corresponding voltage curve placed on the right. The initial state of the Si_8.5_Sn_0.5_Sb-AMBM anode has peaks around 28.2 and 28.8 degree, corresponding to the crystalline Si and SnSb phases, respectively. As lithiation evolves, the SnSb peak rapidly decreases in the subsequent XRD patterns, disappears completely from the fourth XRD curve (around 0.6 V), and does not reappear at the end of delithiation. The crystalline Si peak also greatly reduces with the progress of lithiation and is not recovered during delithiation [54, 55, 68, 69]. Ex-situ XRD tests were carried out on electrodes charged and discharged to different degrees in the second and tenth cycles. As shown in Fig. 3b, no peaks corresponding to crystalline Si and SnSb alloy were observed in these curves. These results indicate that the Si_8.5_Sn_0.5_Sb-AMBM anode exhibits a similar lithiation/delithiation behavior to amorphous Si from the second cycle onward. When pure Si is discharged to ~ 0.05 V, amorphous lithiated Si undergoes a two-phase transformation process to the Li_15_Si_4_ crystalline phase. To avoid stress concentrations caused by the phase transition process, a discharge limit (0.07 to 0.06 V) is usually set for LIBs with Si anodes [37, 54]. But in Fig. 3a, b, when Si_8.5_Sn_0.5_Sb-AMBM anode is discharged to 0.01 V, no peaks corresponding to the Li_15_Si_4_ crystalline phase appear [54]. The characteristic peaks of the crystalline Li_15_Si_4_ phase were also not observed in the first CV curve (Fig. S15). One possible reason is that the lattice distortion induced by the inclusion of Sb and Sn atoms in the Si matrix effectively hinders the formation of long-range ordered structures and suppresses the two-phase reaction. The faint presence of the peak for Si in Fig. 3a at the end of discharge segment may be due to the fact that some of the anode particles coated on the Be window in the in-situ XRD device are not well electrically conducted. Thus, when the voltage is scanned to 0.01 V, these particles haven’t been lithiated and remain the original crystalline Si structure.

The XPS results for the Si_8.5_Sn_0.5_Sb-AMBM and Si_8.5_Sn_0.5_Sb-Mix anodes after 3 cycles at 0.1 A g^−1^ are shown in Figs. S16 and S17, and the chemical compositions of the SEI formed on these two electrodes are very similar. The mechanical properties of SEI including Young’s modulus and elastic strain limit are evaluated by a two-step nanoindentation test based on the atomic force microscope [70–72]. The average values of Young’s modulus and elastic strain limit of the SEI formed on Si_8.5_Sn_0.5_Sb-AMBM electrode are close to those of the SEI formed on Si_8.5_Sn_0.5_Sb-Mix electrode but the range of data distribution is much smaller. These results indicate that by uniformly distributing Sn and Sb within the Si microparticle does not modify the chemical compositions of SEI but facilitates a more homogeneous SEI than just mix the Si, Sn and Sb particles together.

### Morphological Evolution of Anodes

SEM was used to assess the evolution of electrode morphology after cycling. SEM images taken from the Si_8.5_Sn_0.5_Sb-AMBM anodes after 3 pre-cycles at 0.1 A g^−1^ and 47 cycles at 1 A g^−1^ are shown in Fig. S19a, b, respectively. The electrode morphologies are similar in these two images. In marked contrast to Si_8.5_Sn_0.5_Sb-Mix anode (Fig. S20), EDS images for Si_8.5_Sn_0.5_Sb-AMBM anode taken after 3 pre-cycles at 0.1 A g^−1^ (Fig. 3c) and 47 cycles at 1 A g^−1^ (Fig. S21) also indicate uniform distributions of Sb and Sn in Si. In addition, the HRTEM and diffraction results (Fig. 3d) exhibit obvious amorphization features, which are consistent with previous XRD and electrochemical test results. As shown in the SEM results, the anode particle size prepared by our current method has a wide range of distribution, yet great cycling performance is achieved. The preparation technique could be further refined in future work to improve the uniformity of the alloy particle size, which would benefit the battery performance.

**Fig. 4:**
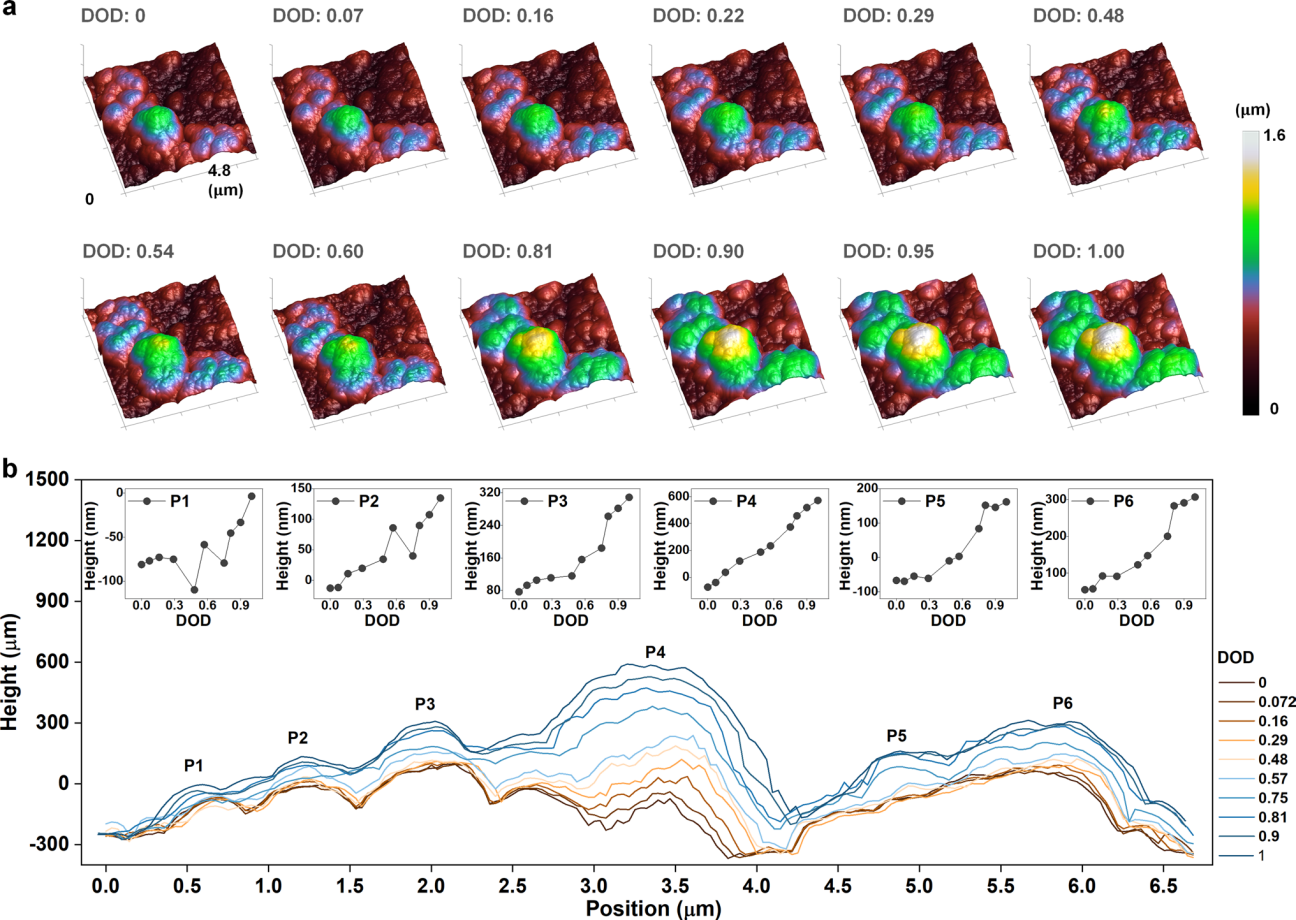
Morphological evolution of anodes. **a** 3D topography images of the Si_8.5_Sn_0.5_Sb-AMBM anode at different depths of discharge during the first cycle. **b** Height profiles corresponding to the slice made from the top-left to the bottom-right of the topography image in Fig. 4a. The insets are the height values of the vertices of marked particles

In-situ AFM tests were performed to visualize the morphological changes of anode particles during cycling. Figure 4a shows the three-dimensional (3D) topography images of the same particle at different depths of discharge (DOD: current capacity/total capacity) during the first cycle. A slice was made from the top-left to the bottom-right in each morphological image of Fig. 4a, and the corresponding height profile is drawn in Fig. 4b. Six particles are marked in the height profiles and the height values of their vertices are plotted above the corresponding particle in Fig. 4b. The height changes of the six particles and the size changes in different directions of each particle are almost synchronized, which is consistent with the isotropic lithiation swelling of amorphous Si. During the entire process, no cracking or pulverization of anode particles were observed. In-situ AFM characterization was also carried out on the Si_8.5_Sn_0.5_Sb-Mix anode. The 3D topography images and height profiles are provided in Fig. S22. Due to the small particle size of commercial powders, a localized region in Fig. S22a is marked with a rectangular box and enlarged in Fig. S22b. As the lithiation process continues, the expansion, fragmentation, and drifting in electrolyte of the anode particle can be clearly seen in the magnified images. In addition, the section height profiles indicate that the volume changes of different particles are not synchronized. It can also be observed that the height of particles P3 and P4 first increase and then drop sharply, suggesting the occurrence of particle pulverization. Moreover, there are some particles whose height hardly change during the discharge process, which probably have lost electrical connections and cannot be lithiated. The comparison of these topographic results provides a compelling evidence that the concentration modulation of Si with Sn and Sb using arc melting and high-energy ball milling can effectively improve the mechanical integrity of micron-sized Si-rich anodes during cycling.

## Discussion

### Finite Element Simulation

To reveal the mechanism of the improved mechanical stability of Si_8.5_Sn_0.5_Sb-AMBM anode, finite element simulations (FEM) were performed on the dimension change, Li concentration distribution, and stress state of individual anode particle during lithiation. In the discharge cycle, lithiation starts from the surface of anode and proceeds inward. A Li concentration gradient forms from the particle surface to the centre, resulting in varying degrees of volume change and stress [73–76]. Simulations were executed with COMSOL Multiphysics to first generate Li concentration distributions and then calculate stresses and strains based on the concentration results (see SI for more details). Thermal effects and the influence of stress on Li diffusion were not considered in the simulations. The mass transport of Li in Si was simulated according to Fick’s second law. A sharp interface between the lithiated and unlithiated regions of Si during lithiation has been widely reported in in-situ TEM results [77, 78]. This sharp interface suggests that, in addition to diffusion, there are other processes that affect the transition from Li-free to lithiated regions, such as the dissociation of Si–Si bonds [79]. A general approach to dealing with this phenomenon in FEM simulations is to employ the concentration-dependent diffusivity of Li (Eq. S4) to simulate the two concurrent processes in a unified manner [79–83]. In short, Li diffuses much faster in the lithiated region than in the unlithiated region. After the Li concentration distribution was generated, the strain was calculated to be proportional to the Li concentration (Eq. S5), and an isotropic expansion coefficient was applied. The anode particle was modelled as a 1/8 sphere to save computational time. Figure S23 shows a schematic diagram of the model along with the boundary conditions. In the simulation, the sphere was allowed to expand freely, and a Li flux corresponding to the charging current of 0.1 A g^−1^ was applied to the outer surface of the sphere (surface 4 in Fig. S23).

**Fig. 5:**
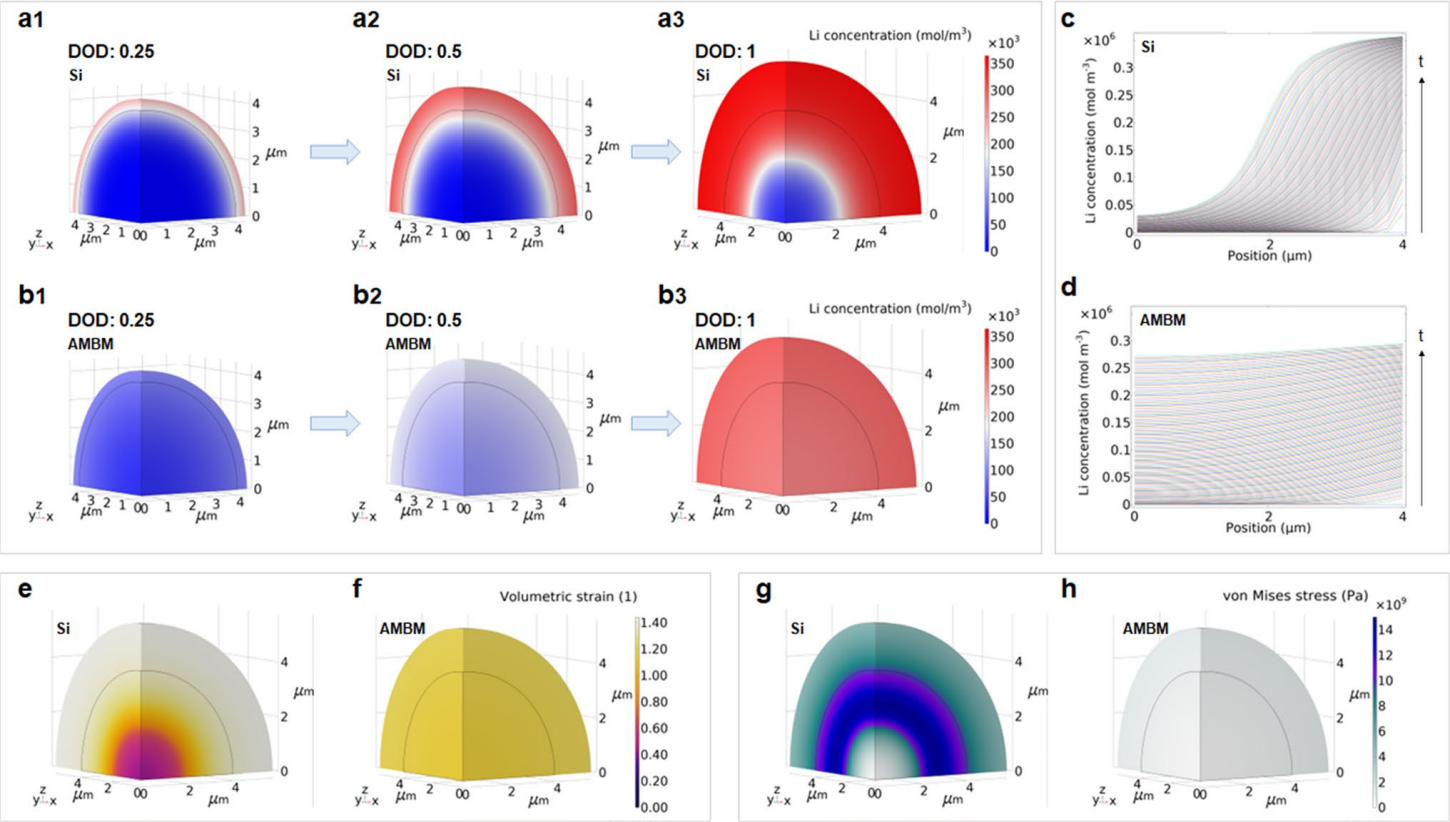
Finite element simulations. The distribution of Li concentration in **a** Si and **b** Si_8.5_Sn_0.5_Sb-AMBM anode at different depths of lithiation. Distribution of Li concentration in **c** Si and **d** Si_8.5_Sn_0.5_Sb-AMBM anodes at different distances from the centre of sphere during lithiation. The distribution of volumetric strain in **e** Si and **f** Si_8.5_Sn_0.5_Sb-AMBM anode when fully lithiated. The distribution of von Mises stress in **g** Si and **h** Si_8.5_Sn_0.5_Sb-AMBM anode when fully lithiated

Figure 5a, b provides the Li concentration profiles of Si and Si_8.5_Sn_0.5_Sb-AMBM anodes at different DODs (0.25, 0.5, and 1.0), respectively. For all DODs, the Li concentration gradient in the Si anode (Fig. 5c) is significantly larger than that in the Si_8.5_Sn_0.5_Sb-AMBM anode (Fig. 5d). These results indicate that Li is rapidly homogenized inside the Si_8.5_Sn_0.5_Sb-AMBM anode, although its diffusivity is only 10 times larger than that of Si at the same Li concentration in the simulation parameter settings. In contrast, Li takes longer time to reach the centre of Si anode and exhibits a distinct phase boundary between the Li-rich and Li-poor regions. Consequently, the volumetric strain of Si anode varies greatly at different distances from the sphere centre (Fig. 5e) but is fairly uniform for the Si_8.5_Sn_0.5_Sb-AMBM anode (Fig. 5f). Compared to the Si_8.5_Sn_0.5_Sb-AMBM anode, the uneven lithiation swelling of Si anode results in higher Von Mises stress levels (Figs. 5g, h and S24) and a greater propensity for mechanical failure. To eliminate the concern that the difference in stress levels is caused by the difference in specific capacity, Fig. S25 compares the Li concentration and stress results for the two anodes at 1 Ah g^−1^, and similar phenomena were observed.

We also simulated the conditions that Sn and Sb are incorporated into the Si particle as discernible bars or spheres. The Li concentration results in Fig. S26 indicate that the inclusion of Sn and Sb enhances the Li diffusion compared to pure Si anode. However, severe stress concentrations appear at the Sn–Si and Sb–Si interfaces. The maximum von Mises stress within the entire 1/8 sphere is plotted against the lithiation time in Fig. S27. The Si_8.5_Sn_0.5_Sb-AMBM sample has the lowest maximum stress value throughout the lithiation process, while the maximum stress value of Si anodes with discernible Sn and Sb inclusions is even higher than that of pure Si anode. These results validate the necessity of the high-energy ball milling step in forming the Si_8.5_Sn_0.5_Sb-AMBM sample, which greatly weakens the phase boundary among Si, Sn, and Sb and reduces the risk of stress concentration. In conclusion, the simulation results corroborate our proposed stress relief mechanism. To improve the mechanical stability of Si-based microparticle anodes, it is necessary to increase the Li diffusivity while avoiding stress concentrations.

### Extension to Four and Five Element Alloy Systems

**Fig. 6:**
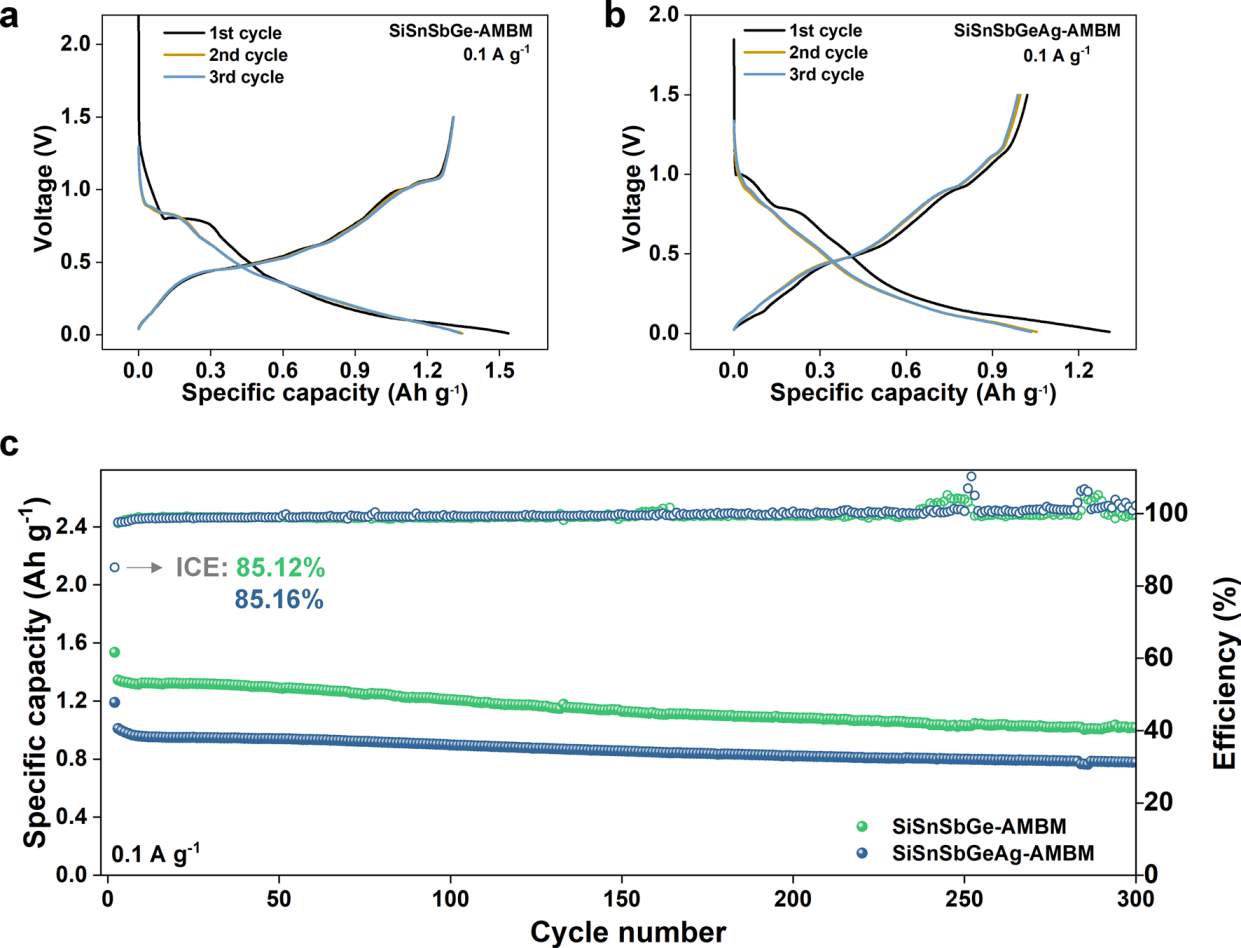
Extension to four and five element alloy systems. The first three cycles of galvanostatic curves of **a** SiSnSbGe-AMBM (0.94 mg cm^−2^) and **b** SiSnSbGeAg-AMBM (2.35 mg cm^−2^) anode at 0.1 A g^−1^. **c** The performance of SiSnSbGe-AMBM (0.94 mg cm^−2^) and SiSnSbGeAg-AMBM (1.57 mg cm^−2^) anodes at 0.1 A g^−1^ over 7 month cycling

To test the applicability of the stress mitigation mechanism, we prepared micron-scale Si-based ternary alloy anode with a composition of Si_4_Sn_2.8_Sb (Figs. S28–S31), as well as quaternary and pentanary alloy-type anodes for LIBs using the same technique. The discharge capacities of the Si_4_Sn_2.8_Sb-AMBM microparticle anode after 300 cycles at 1.0 and 3.0 A g^−1^ are 0.81 and 0.68 Ah g^−1^, respectively, corresponding to a retention rate of 80.6% and 86.2%, respectively, relative to the capacity of the first cycle after activation. The quaternary system includes Si, Sb, Sn and Ge and the pentanary system adds Ag on this basis. These two anodes are referred to as SiSnSbGe-AMBM and SiSnSbGeAg-AMBM, respectively. In both systems, the mole fraction of each element is equal. The microparticle SiSnSbGe-AMBM and SiSnSbGeAg-AMBM anodes demonstrate uniform element distribution (Figs. S32–S33) and extraordinary cycling stability. Figure 6a, b shows the first three cycles of voltage curves for the SiSnSbGe-AMBM and SiSnSbGeAg-AMBM anode, respectively. A slight voltage plateau can be observed in the first cycle, and a smooth sloped curve takes place from the second cycle, indicating that its charging/discharging process is closer to a solid solution reaction than a two-phase reaction [58]. EDS images of SiSnSbGeAg-Mix (Fig. S34a) and SiSnSbGeAg-AMBM (Fig. S34b) anode taken after 3 cycles at 0.1 A g^−1^ and 47 cycles at 1 A g^−1^ also show that the distribution of the various elements in the electrodes remains uniform.

The long-cycle performance of SiSnSbGe-AMBM and SiSnSbGeAg-AMBM anodes at 0.1 and 1 A g^−1^ are shown in Figs. 6c and S35, respectively. Both anodes yield high initial coulombic efficiencies of over 80% thanks to the low surface area of microsized particles. The discharge capacities of the SiSnSbGe-AMBM and SiSnSbGeAg-AMBM anodes after 300 cycles at 0.1 A g^−1^ are 1.02 and 0.78 Ah g^−1^, respectively. In addition, after 1000 cycles at 1 A g^−1^, the capacity retention of the SiSnSbGeAg-AMBM anode is as high as 79.43% relative to the first cycle after activation, corresponding to an extremely small capacity decay rate of 0.02% per cycle. The superior cycling stability of SiSnSbGe-AMBM and SiSnSbGeAg-AMBM microparticle anodes proves that the arc melting together with high-energy ball milling is a promising route to prepare microparticle alloy-type anodes for LIBs.

## Conclusions

For micron-sized Si anodes, severe particle pulverization during cycling hinders their practical application in LIBs. Our work demonstrates that eliminating high stressors that lead to particle fracture can effectively improve the cycling stability of Si-based microparticle anodes. To maintain the competitive advantages of high specific capacity and low cost of Si anode, Sn and Sb with the same advantages are used as modifiers. Combining arc melting and high-energy ball milling, Sn and Sb are uniformly distributed inside the Si microparticles. The prepared Si_8.5_Sn_0.5_Sb microparticle anode has a much higher electronic conductivity and Li diffusivity than Si, which facilitates the reduction of stresses caused by Li concentration gradients. Moreover, the electrode process of Si_8.5_Sn_0.5_Sb microparticle anode is similar to isotropic solid solution reactions, which greatly avoids the stress concentration caused by the two-phase reactions. Under these mechanisms, the discharge capacities of Si_8.5_Sn_0.5_Sb microparticle anode after 100 cycles at 1.0 and 3.0 A g^−1^ are 1.62 and 1.19 Ah g^−1^, respectively, corresponding to a retention rate of 94.2% and 99.6%, respectively, relative to the capacity of the first cycle after activation. We believe that this stress regulating mechanism enabled by the simple industry-compatible fabrication method combining arc melting and high-energy ball milling could present tremendous opportunities for the impending low-cost and high-energy density Li ion batteries.

### Supplementary Information

Below is the link to the electronic supplementary material.

Supplementary file1 (PDF 3597 kb)
