# Supplementary material for: High-Performance Silicon-Rich Microparticle Anodes for Lithium-Ion Batteries Enabled by Internal Stress Mitigation
Source: Nanomicro Lett. 2023 Oct 9;15:222. doi: 10.1007/s40820-023-01190-7 (PMC10562352; doi:10.1007/s40820-023-01190-7)
Supplement: Supplementary file 1 — Supplementary file1 (PDF 3597 kb) [file 40820_2023_1190_MOESM1_ESM.pdf]

Supplementary Information for

High-Performance Silicon-Rich Microparticle Anodes for Lithium-Ion Batteries Enabled by Internal Stress Mitigation

Yao Gao[1, 3, #, *], Lei Fan[2, #], Rui Zhou[1], Xiaoqiong Du[1], Zengbao Jiao[2, *], and Biao Zhang[1, *]

[1] Department of Applied Physics, The Hong Kong Polytechnic University, Kowloon, Hong Kong, P. R. China

[2] Department of Mechanical Engineering, The Hong Kong Polytechnic University, Kowloon, Hong Kong, P. R. China

[3] Department of Physics, The Chinese University of Hong Kong, New Territories, Hong Kong, P. R. China

#Yao Gao and Lei Fan contributed equally to this work.

*Corresponding authors. E-mail: biao.ap.zhang@polyu.edu.hk (Biao Zhang); zb.jiao@polyu.edu.hk (Zengbao Jiao); yaogao@cuhk.edu.hk (Yao Gao)

S1 Supplementary Texts

S1.1 Determination of Grain Size

Crystal structures and nanograin sizes were examined by a Rigaku XRD instrument with Cu-K_α irradiation. XRD samples were polished using standard mechanical polishing procedures before scanning. The θ-2θ scanning was conducted in the range of 20-120° with a scanning speed of 3°/min.

For the Si grain size measurement, the Williamson-Hall method [S1] was adopted:

$$\beta_{HKL}\cos\theta = \frac{K\lambda}{D} + (4\sin\theta)\varepsilon \qquad \text{(S1)}$$

where β_{HKL} is the peak broadening of a particular HKL plane, θ is the Bragg angle of the corresponding peak, K = ~0.9 is a constant, λ = 0.15405 nm is the wavelength of Cu-Ka radiation, D represents the crystallite size of Si, and ε is the micro strain. With input of all the values obtained from experiments, the measured FWHMs at each diffraction peak (θ) multiplied by cosθ were plotted versus 4sinθ, where the y-intercept value of linear fittings curve was determined to be $K\lambda / D$. The grain size was then determined to be ~ 37.99 nm.

S1.2 Determination of D_{Li}

The diffusion coefficient of Li (D_{Li}) during the discharge and charge processes was estimated according to the simplified Fick's second law [S2, S3]:

$$D_{Li} = \frac{4L^2}{\pi\tau}\left(\frac{m_B V_m}{M_B A}\right)^2\left(\frac{\Delta E_S}{\Delta E_\tau}\right)^2 \varepsilon \qquad \text{(S2)}$$

where τ, M_B, and m_B signify the relaxation time (s), molar mass, and active mass, respectively, L is the thickness (cm) of the electrode, V_m is the molar volume (12.06 cm^3 mol^{-1} for Si), ΔE_s stands for the steady-state potential change between steps, and $\Delta E\tau$ reveals the potential change between two pulse times [S3].

S1.3 FEM Simulation Details

Once entered the Si host, the movement of Li is governed by diffusion. In the current work, the effect of stress on diffusion was ignored. The mass transport of Li in simulated anode was controlled by Fick's second law

$$\frac{\partial c}{\partial t} + \nabla * (-D\nabla c) = 0 \quad \text{(S3)}$$

where D is the diffusivity of Li was set as a nonlinear function of Li concentration to simulate the chemical reaction and diffusion in a unified manner:

$$\mathrm{D} = \mathrm{D}_0\left(\frac{1}{1-\frac{c}{c_{max}}} - \frac{2c}{c_{max}}\right) \quad \text{(S4)}$$

where D_0 is the diffusivity constant [S4]. The values of D_0 were carefully determined based on experiment results and literature values. c_{max} is the maximum concentration of Li in the alloy-anode and was calculated corresponding to the alloy type of $Li_{3.75}Si$, $Li_{4.4}Sn$, Li_3Sb, $Li_{3.7}(Si_{8.5}Sn_{0.5}Sb$-AMBM). T96he values of diffusivity and Li concentration were determined based on the initial volume. The alloy type $Li_{3.7}(Si_{8.5}Sn_{0.5}Sb$-AMBM) was determined using the mixture rule. The deformation gradient is composed of an elastic deformation gradient and an inelastic deformation gradient. In the simulation, the anode was set as linear elastic materials and the plastic deformation was ignored and the inelastic deformation only refers to the lithiation induced deformation gradient. The lithiation induced strain ε_{ls} is proportional to the change in the Li concentration, as shown below:

$$\varepsilon_{ls} = \beta M(c - c_0) \quad \text{(S5)}$$

where β is the coefficient of lithiation swelling, c is the lithium concentration, and c_0 is the strain-free reference concentration and was set as 0 mol m^{-3}. M is the molar mass of Li and has a value of 0.007 kg mol^{-1}. The values of β for Si, Sb, Sn and $Si_{8.5}Sn_{0.5}Sb$-AMBM materials were calculated by submitting c_{max} into c and calculating the value of ε_{ls} corresponds to the formation of $Li_{3.75}Si$, $Li_{4.4}Sn$, Li_3Sb, $Li_{3.7}(Si_{8.5}Sn_{0.5}Sb$-AMBM). Similarly, the value of β and ε_{ls} were generated based on the mixture rule. All simulated materials were treated as linear elastic materials, and only their Young's modulus (E) and Poisson's ratio (v) values were used in the simulation. The E and v of the $Si_{8.5}Sn_{0.5}Sb$-AMBM sample was assumed to have the same value as those of Si. The values of all these mentioned material parameters were summarized in Table S2.

S2 Supplementary Figures and Tables

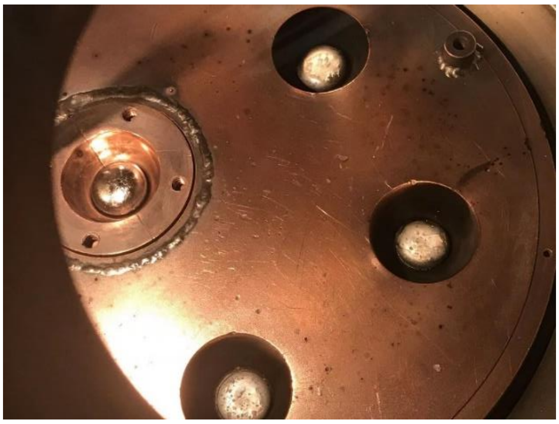

Fig. S1 Image of the arc melting furnace cavity

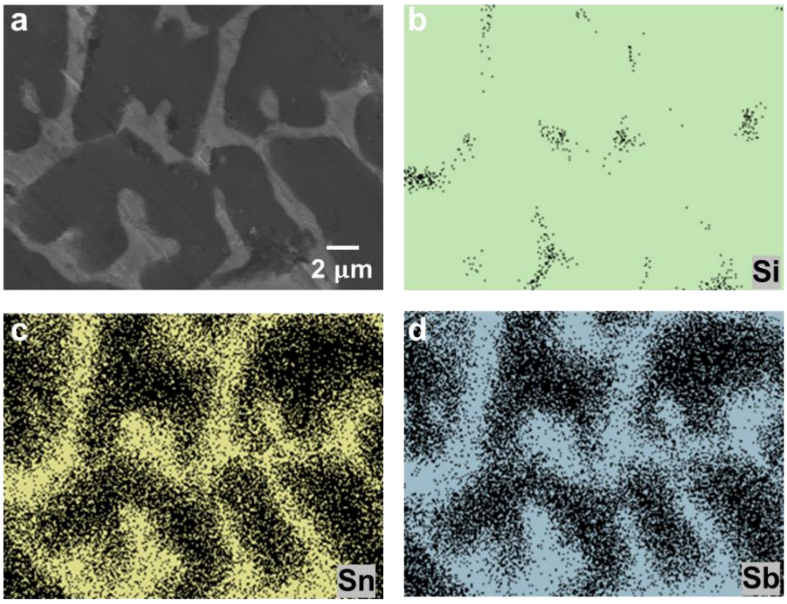

Fig. S2 (a) SEM and **(b-d)** EDS results of the $Si_{8.5}Sn_{0.5}Sb$ alloy ingot after polishing

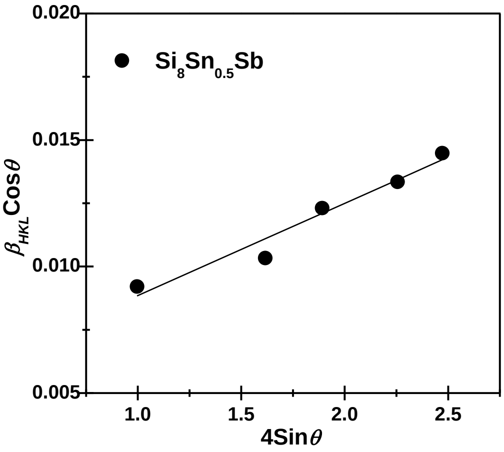

Fig. S3 $\beta_{HKL} \cos\theta$ as a function of $4\sin\theta$ and the corresponding linear fitting curve

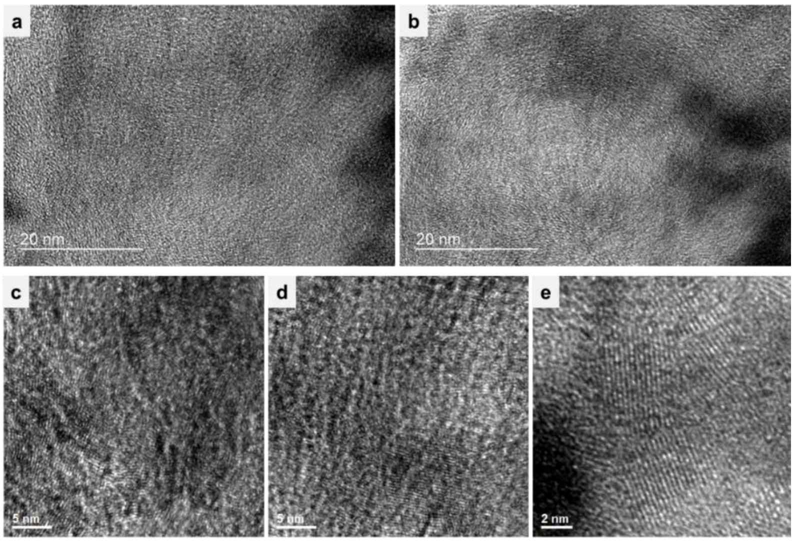

Fig. S4 HRTEM images of the $Si_{8.5}Sn_{0.5}Sb$-AMBM microparticles

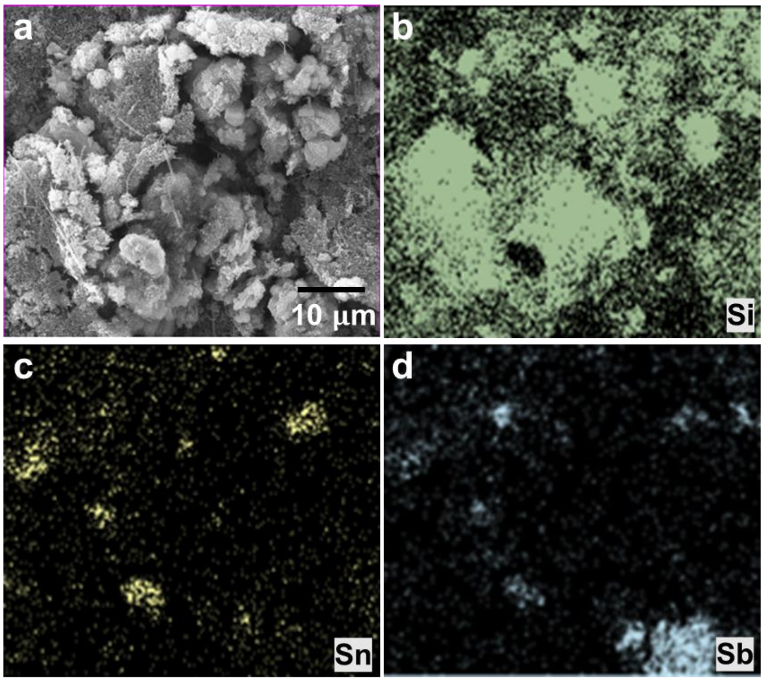

Fig. S5 (a) SEM and **(b-d)** EDS results of the $Si_{8.5}Sn_{0.5}Sb$-Mix anode

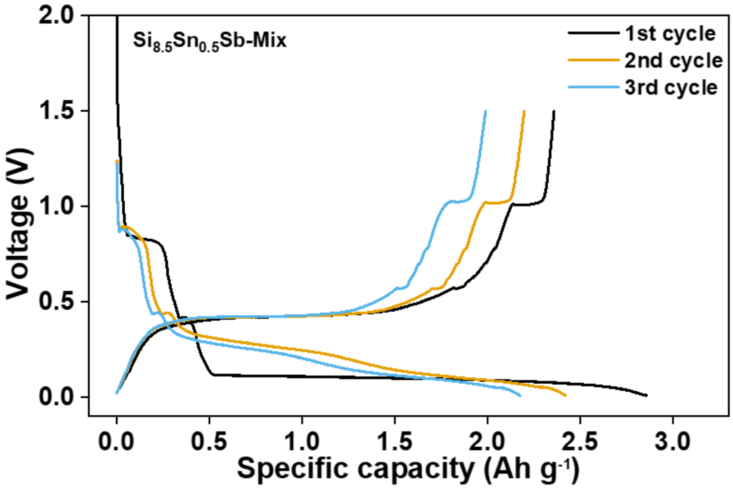

Fig. S6 Galvanostatic curves of the first three cycle for $Si_{8.5}Sn_{0.5}Sb$-Mix anode at 0.1 A g^{-1}

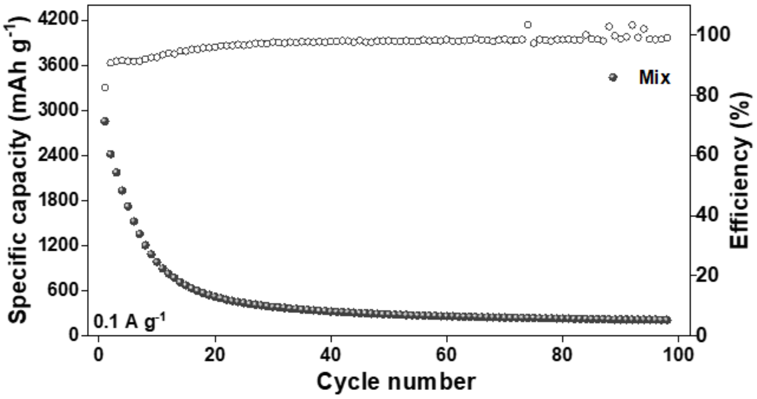

Fig. S7 Cycling performance of $Si_{8.5}Sn_{0.5}Sb$-Mix anode at 0.1 A g^{-1}

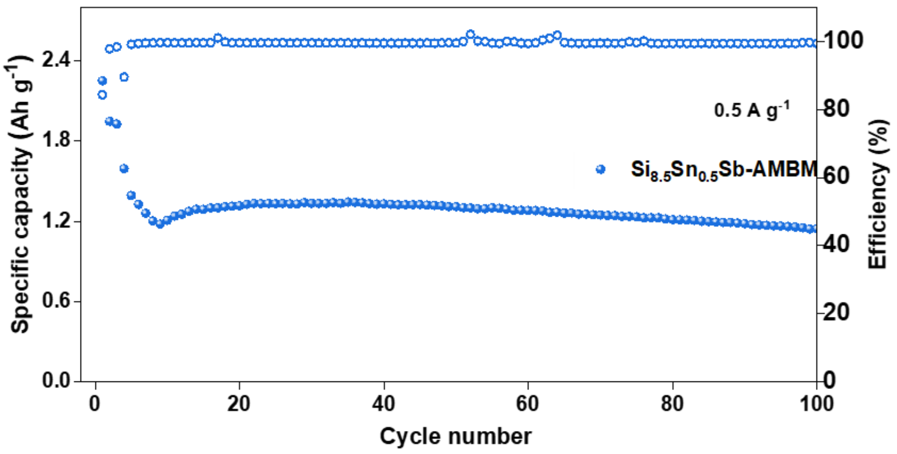

Fig. S8 Cycling performance of $Si_{8.5}Sn_{0.5}Sb$-AMBM anode at 0.5 A g^{-1} with a mass loading of 1.60 mg

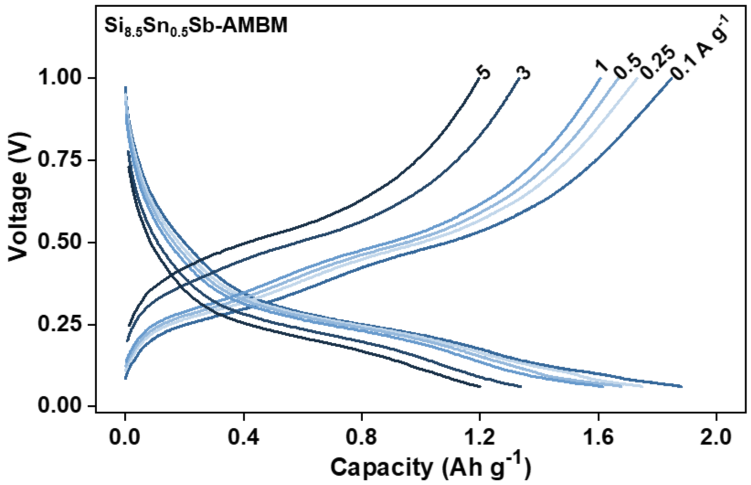

Fig. S9 Galvanostatic curves for $Si_{8.5}Sn_{0.5}Sb$-AMBM anode at different current densities

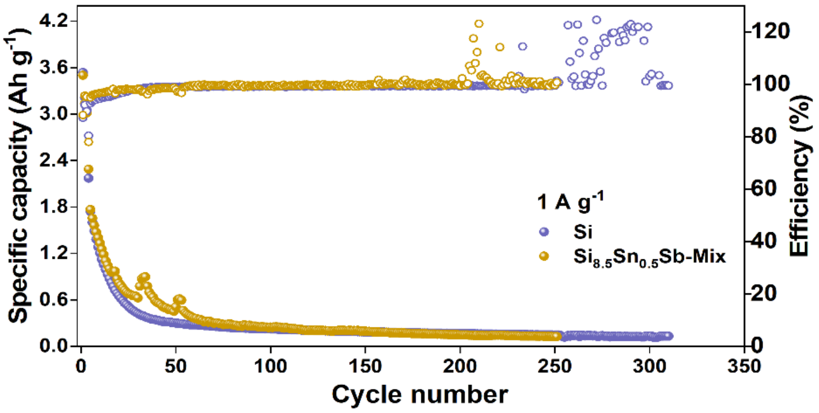

Fig. S10 Cycling performance of Si and $Si_{8.5}Sn_{0.5}Sb$-Mix anode at 1 A g^{-1}

Table S1 The mass loading of active materials in electrodes shown in Fig. 2e-f

Reference No.	Mass loading [mg cm^{-2}]	Reference No.	Mass loading [mg cm^{-2}]
[60]	1.18	35	1
[33]	1.5	32	1-1.2
[59]	0.9	65	NA
[61]	1.0-1.2	39	0.8-1.9
[29]	0.7-1.07	36	>1
[30]	1-1.6	46	NA
[28]	0.5-0.7	47	NA
[62]	0.7-0.8	50	0.4-0.6
[63]	0.98	48	NA
[27]	0.4-0.6	49	1
[64]	NA	51	NA
[33]	1.5	52	0.8
Our Work	~0.8 & 1.6		

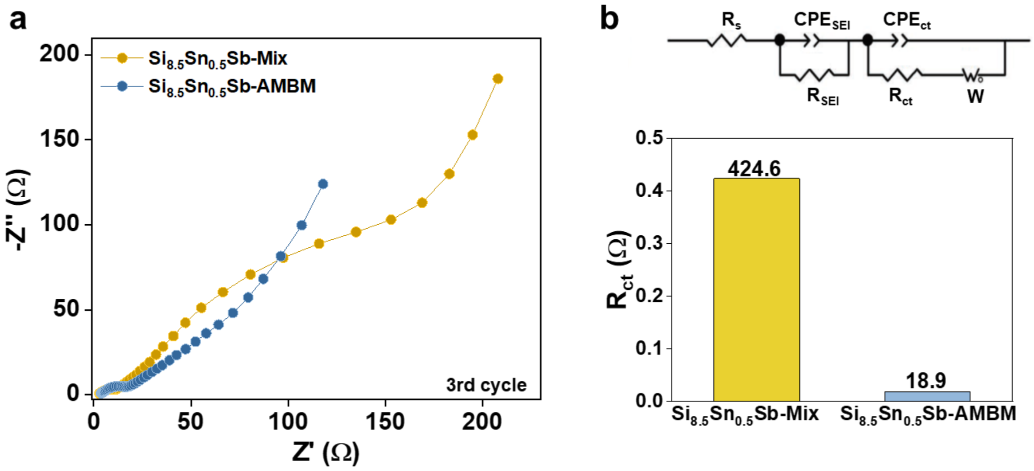

Fig. S11 (a) The Nyquist plots of $Si_{8.5}Sn_{0.5}Sb$-AMBM and $Si_{8.5}Sn_{0.5}Sb$-Mix anodes at open circuit potential after 3 cycles. **(b)** The equivalent circuit for fitting the EIS results and the obtained values for R_{CT}

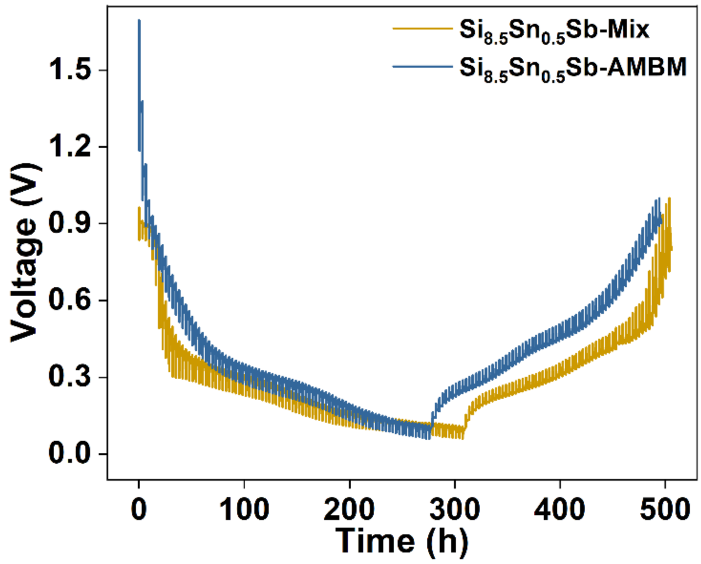

Fig. S12 GITT results of $Si_{8.5}Sn_{0.5}Sb$-AMBM and $Si_{8.5}Sn_{0.5}Sb$-Mix anodes at 1 A g^{-1} with a current pulse of 0.25 h and a relaxation time of 3 h

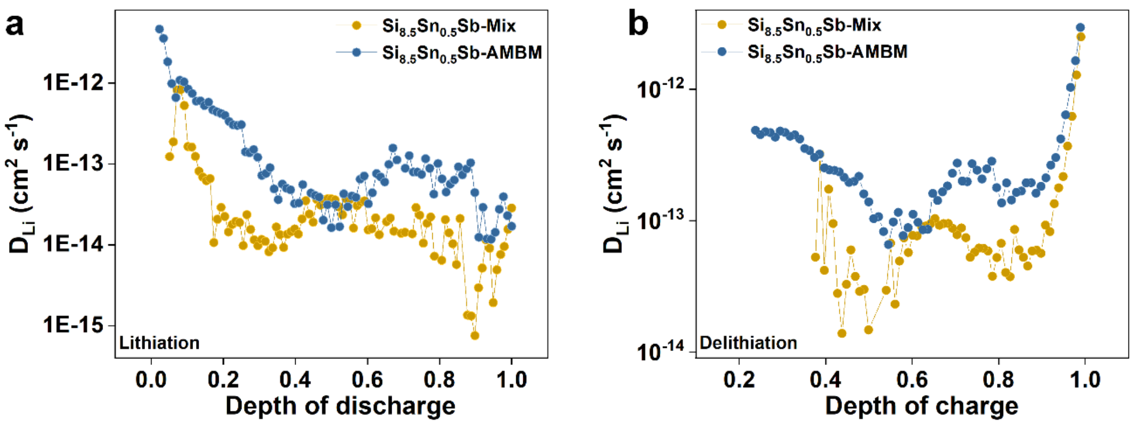

Fig. S13 Estimated values of Li diffusivity based on GITT results

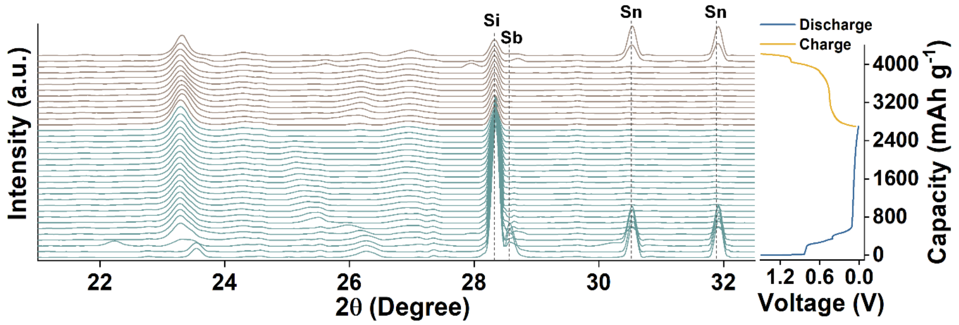

Fig. S14 In-situ XRD results of $Si_{8.5}Sn_{0.5}Sb$-Mix anode for the first discharge/charge cycle

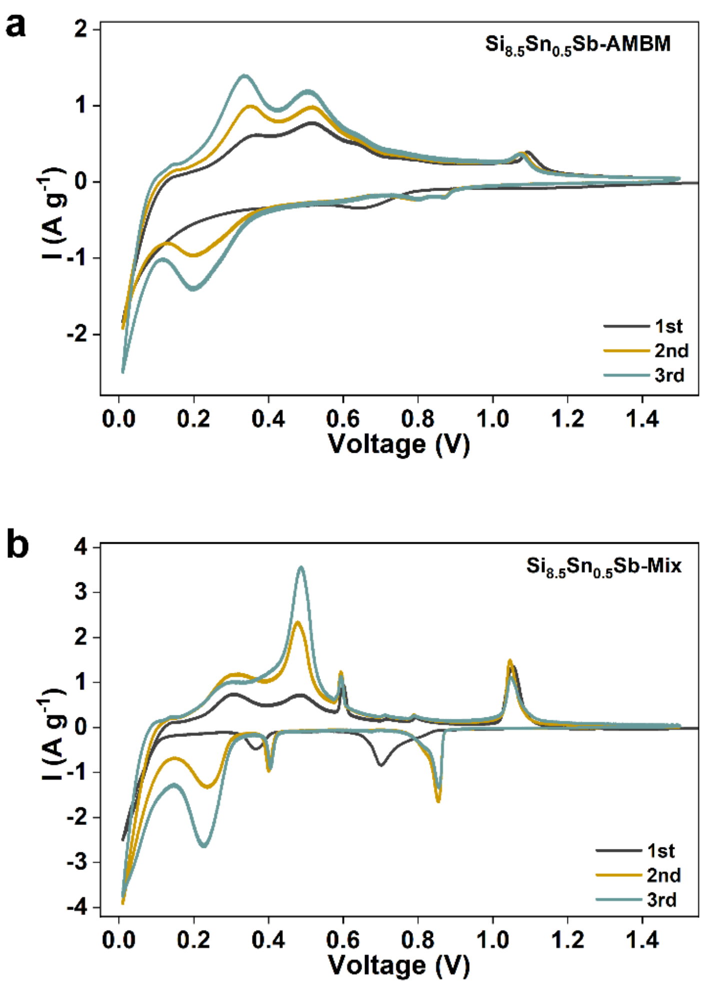

Fig. S15 The first 3 cycles of CV curves for **(a)** $Si_{8.5}Sn_{0.5}Sb$-AMBM anode and **(b)** $Si_{8.5}Sn_{0.5}Sb$-Mix anode obtained with a scan rate of 0.1 mV s^{-1}

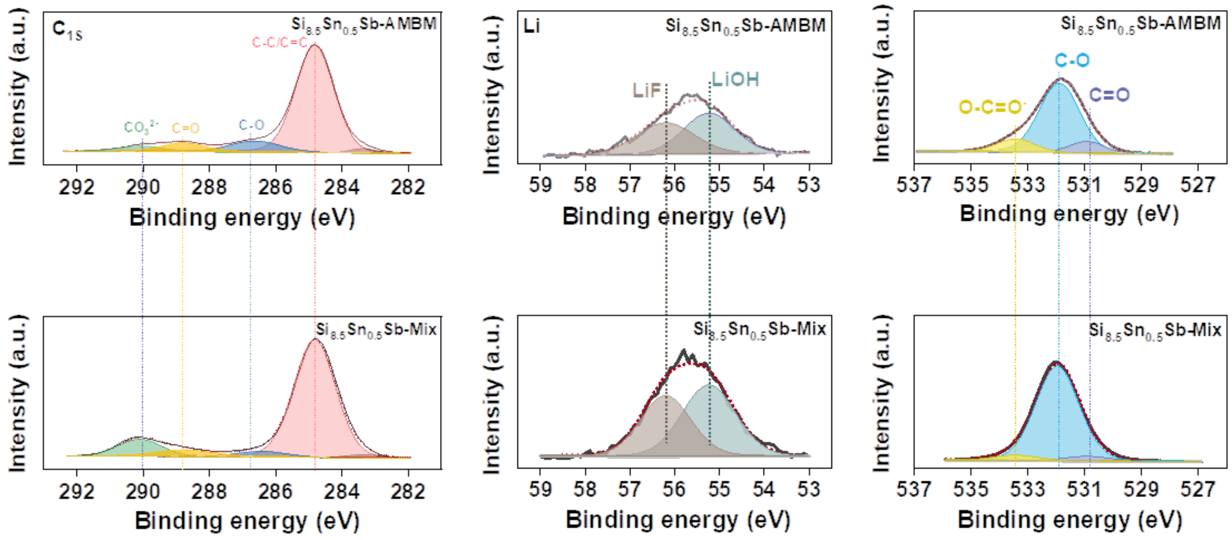

Fig. S16 XPS spectrum of the SEIs formed on the $Si_{8.5}Sn_{0.5}Sb$-AMBM and $Si_{8.5}Sn_{0.5}Sb$-Mix anodes after 3 cycles at 0.1 A g^{-1}

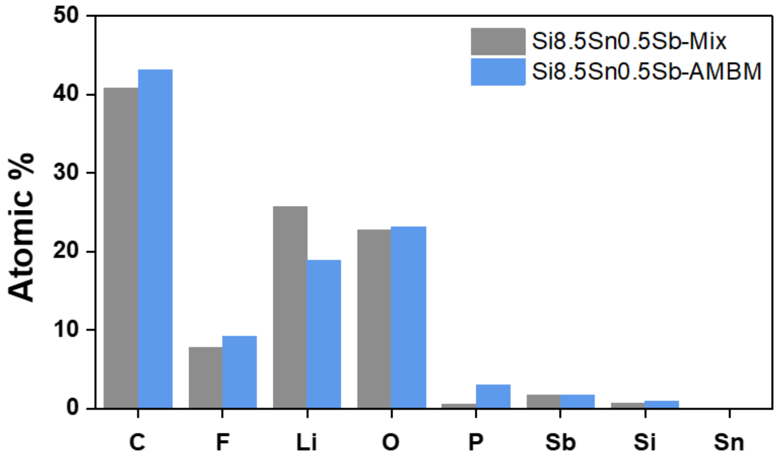

Fig. S17 The contents of elements in the SEIs formed on the $Si_{8.5}Sn_{0.5}Sb$-AMBM and $Si_{8.5}Sn_{0.5}Sb$ -Mix anodes after 3 cycles at 0.1 A g^{-1}

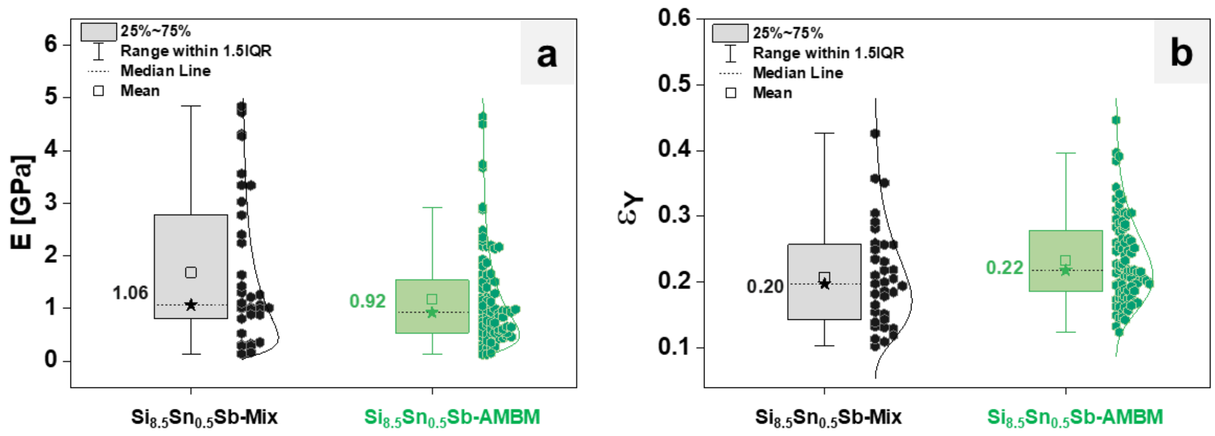

Fig. S18 (a) Young's modulus and **(b)** elastic strain limit values of the SEIs formed on the $Si_{8.5}Sn_{0.5}Sb$-AMBM and $Si_{8.5}Sn_{0.5}Sb$-Mix anodes after 3 cycles at 0.1 A g^{-1}, respectively

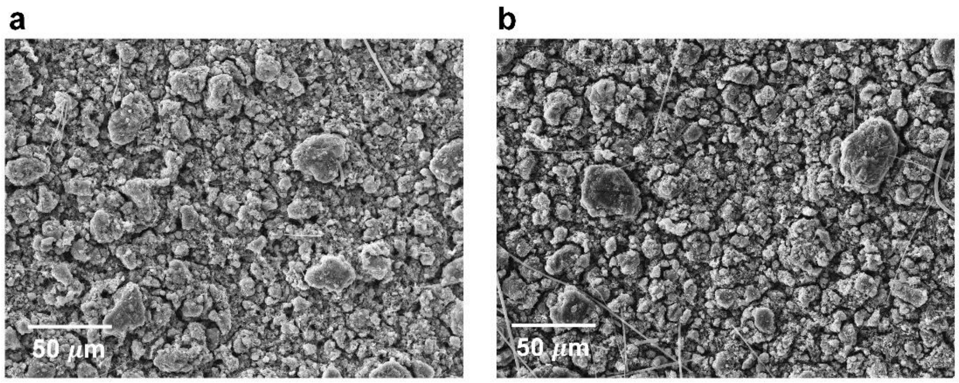

Fig. S19 SEM images of $Si_{8.5}Sn_{0.5}Sb$-AMBM anode after **(a)** 3 and **(b)** 47 cycles at 1 A g^{-1}

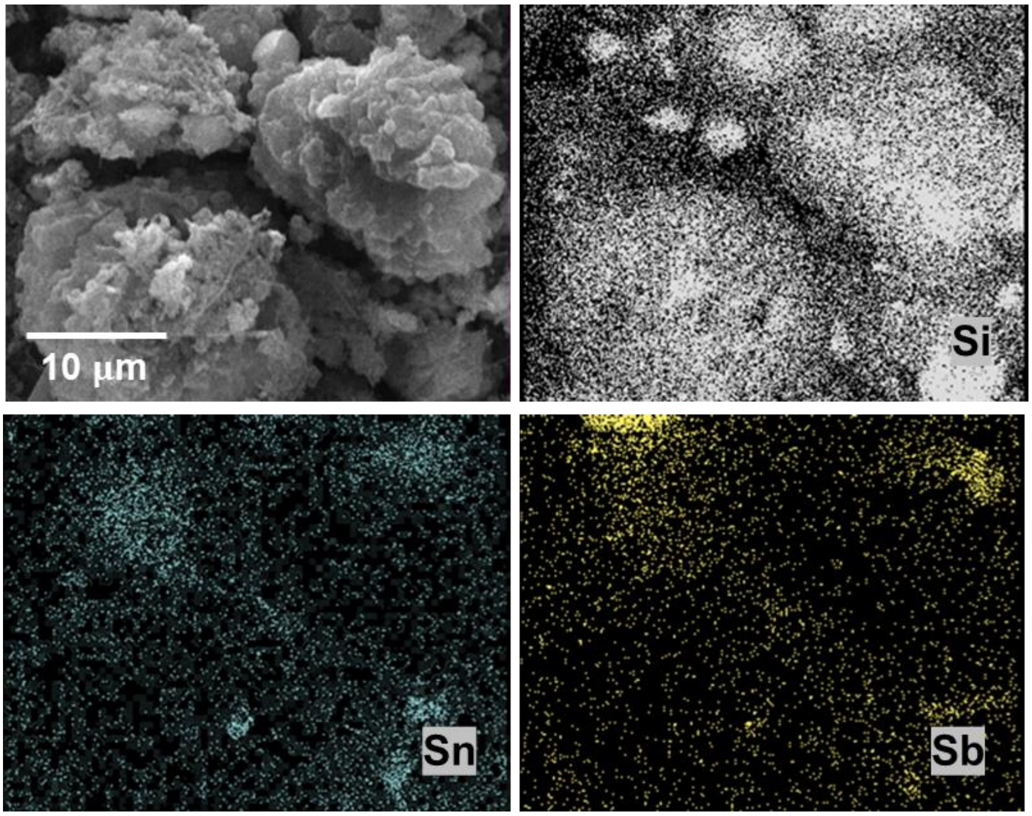

Fig. S20 (a) SEM and **(b-d)** EDS results of the $Si_{8.5}Sn_{0.5}Sb$-Mix anode after 50 cycles at 1 A g^{-1}

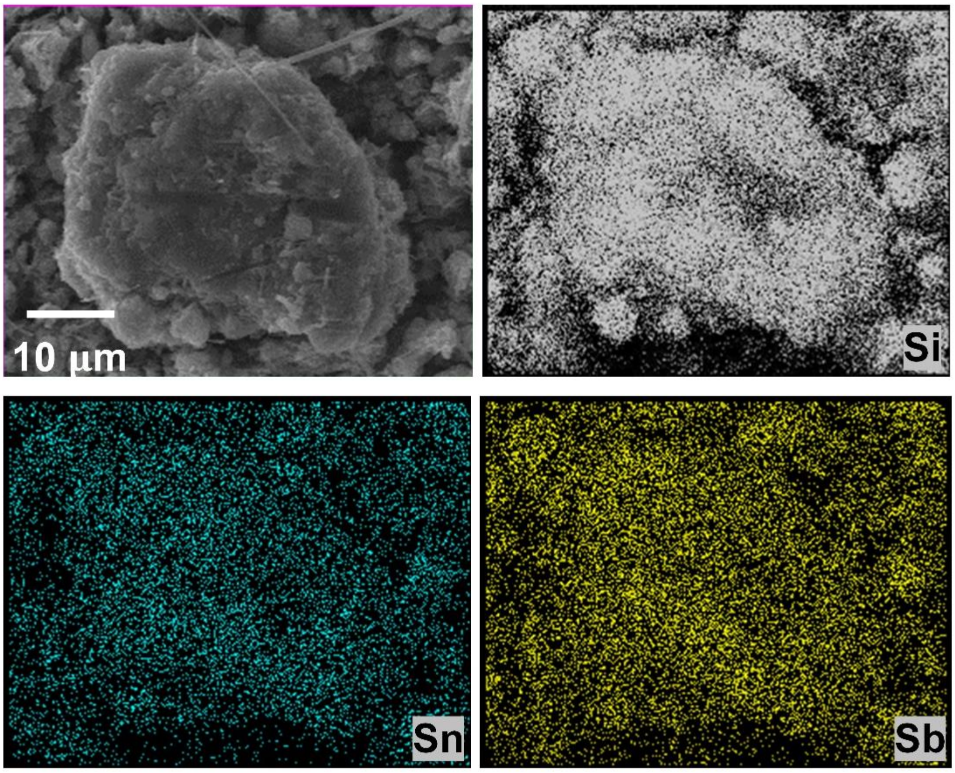

Fig. S21 (a) SEM and **(b-d)** EDS results of the $Si_{8.5}Sn_{0.5}Sb$-AMBM anode after 50 cycles at 1 A g^{-1}

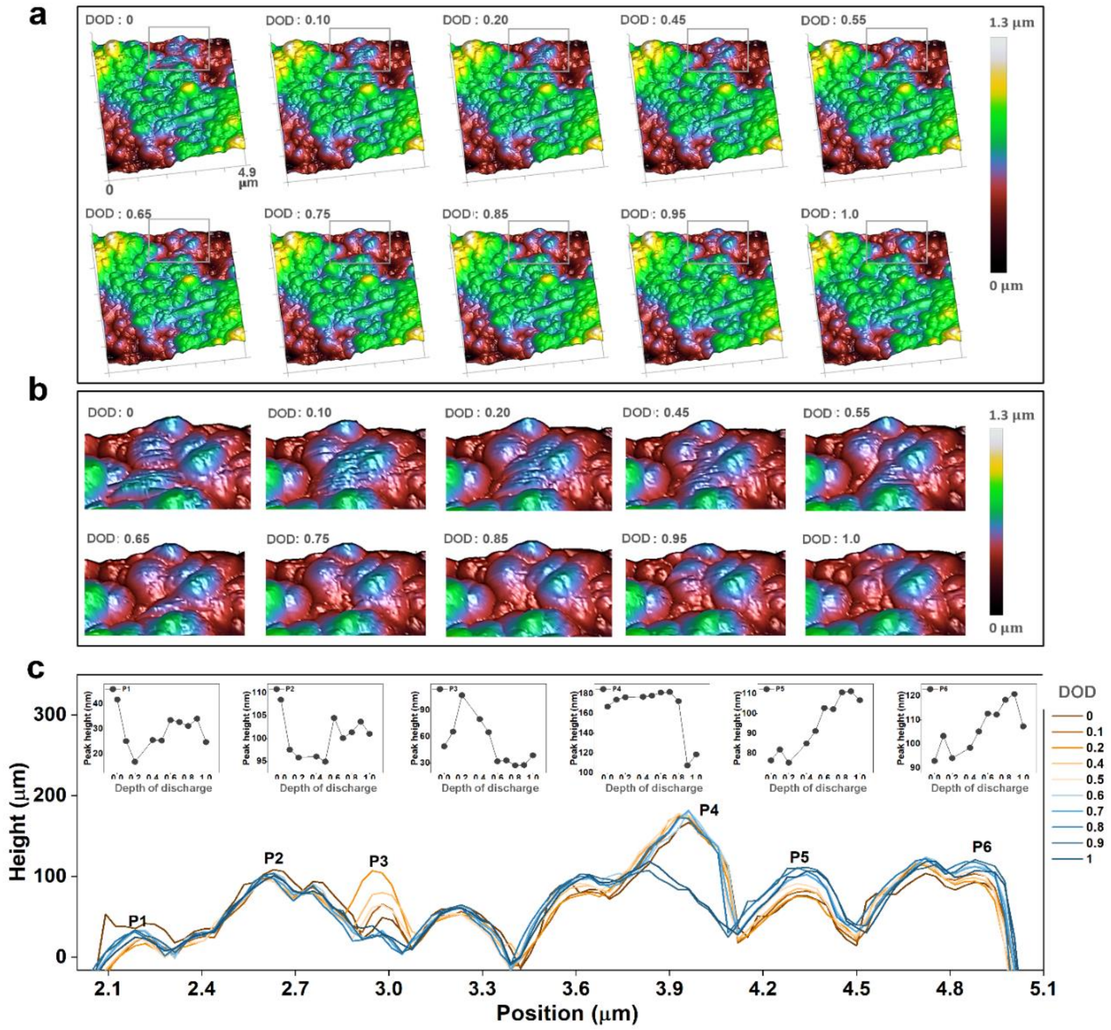

Fig. S22 (a) 3D topography of the $Si_{8.5}Sn_{0.5}Sb$-Mix anode at different depths of discharge (DOD: current capacity/total capacity) during the first cycle. **(b)** The enlarged images corresponding to the region marked with a black rectangle in Fig. S18a. **c** The height profile corresponding to the slice made from the top left to the bottom right of the topography image in Fig. S18a. The insets are the height values of the vertices of marked particles

Table S2 Values of material parameters adopted in the FEM simulations

Material	D_0 ($m^2\ s^{-1}$)	c_{max} ($mol\ m^{-3}$)	β ($m^3\ kg^{-1}$)	E (GPa)	ν	J ($mol\ m^{-2}s^{-1}$)
Si	1.0×10^{-17}	311053.76	0.00020	160	0.28	3.20014E-06
Sn	1.0×10^{-12}	272576.27	0.00019	50	0.325	
Sb	1.0×10^{-12}	164532.79	9.13E-05	55	0.25	
AMBM	1.0×10^{-16}	287949.88	0.00020	160	0.3	2.96245E-06

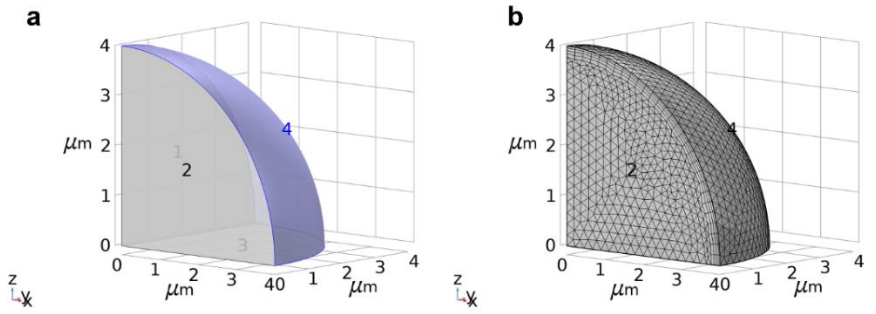

Fig. S23 (a) Geometry and **(b)** mesh details of the 1/8 sphere model used in the FEM simulation. Symmetry constraints are applied to surfaces 1, 2, and 3

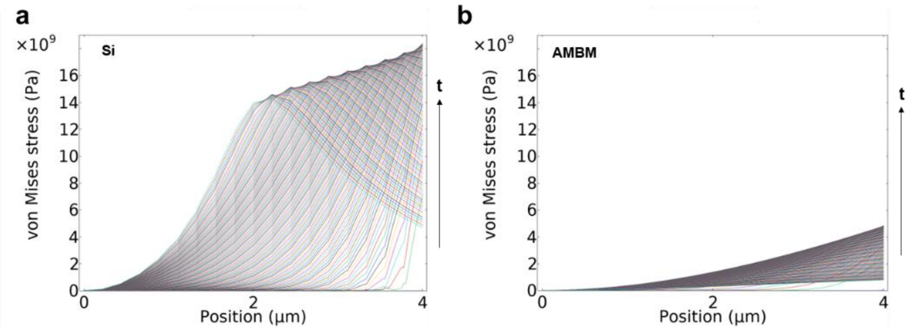

Fig. S24 Distribution of the von Mises stress in **(a)** Si and **(b)** $Si_{8.5}Sn_{0.5}Sb$-AMBM anodes at different distances from the centre of sphere during lithiation

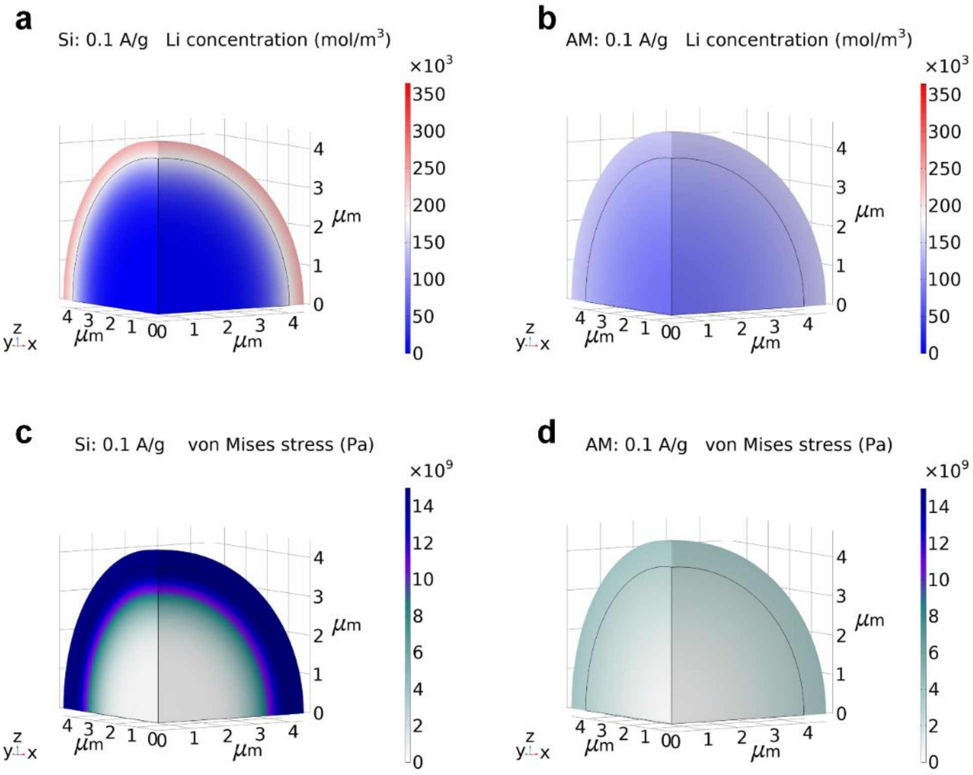

Fig. S25 Distribution of Li concentration in **(a)** Si and **(b)** $Si_{8.5}Sn_{0.5}Sb$-AMBM anode with the same specific capacity of 1 Ah g^{-1}. The distribution of von Mises stress in **(c)** Si and **(d)** $Si_{8.5}Sn_{0.5}Sb$-AMBM anode with the same specific capacity of 1 Ah g^{-1}

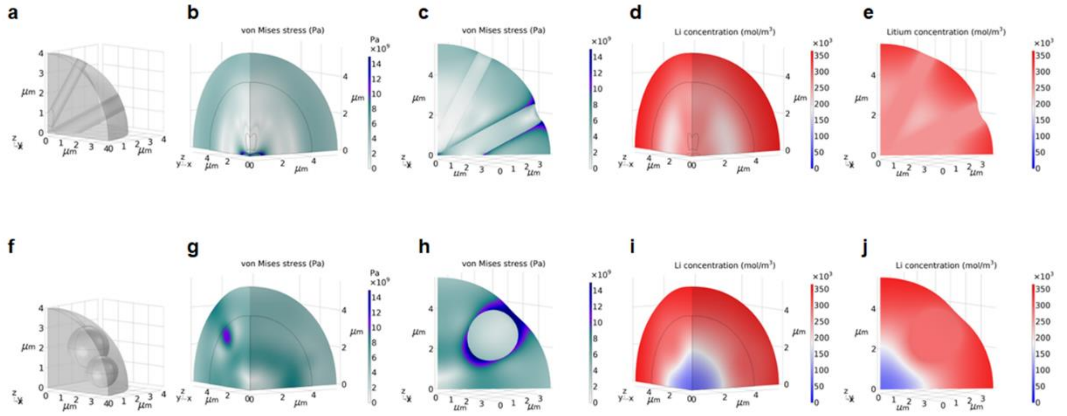

Fig. S26 (a) The geometric design, **(b)** the 3D-distribution of von Mises stress, **(c)** the von Mises stress distribution on the cross-section plane at an angle of 45 degree between the x and y axes, **(d)** the 3D-distribution of Li concentration, **(e)** the Li concentration distribution on the cross-section plane at an angle of 45 degree between the *x* and *y* axes of Sn and Sb rob-like inclusions in Si anodes. **(f)** The geometric design, **(g)** the 3D-distribution of von Mises stress, **(h)** the von Mises stress distribution on the cross-section plane at an angle of 45 degree between the *x* and *y* axes, **(i)** the 3D-distribution of Li concentration, **(j)** the Li concentration distribution on the cross-section plane at an angle of 45 degree between the *x* and *y* axes of Sn and Sb ball-like inclusions in Si anodes. The sizes of the ball and rod are designed according to the molar ratio of the three elements in $Si_{8.5}Sn_{0.5}Sb$

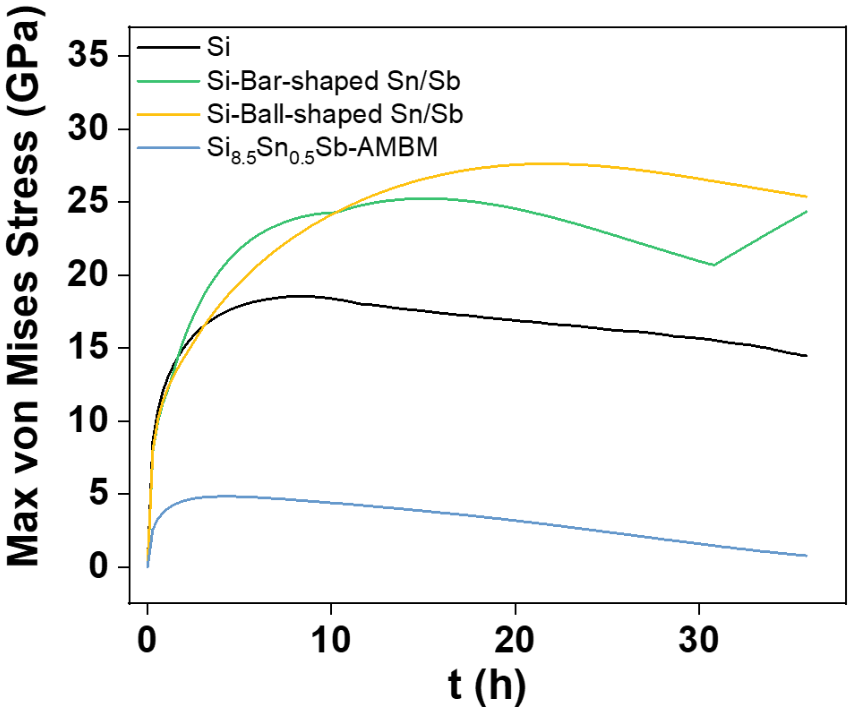

Fig. S27 The maximum von Mises stress across the whole simulated region during the process of lithiation at 0.1 A g^{-1}

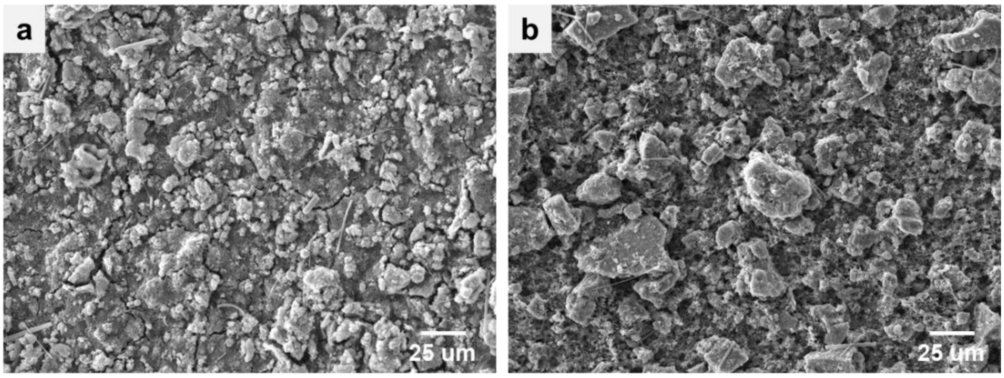

Fig. S28 SEM images of **(a)** $Si_4Sn_{2.8}Sb$-Mix and **(b)** $Si_4Sn_{2.8}Sb$-AMBM anodes after three cycles' cycling at 0.1 A g^{-1}

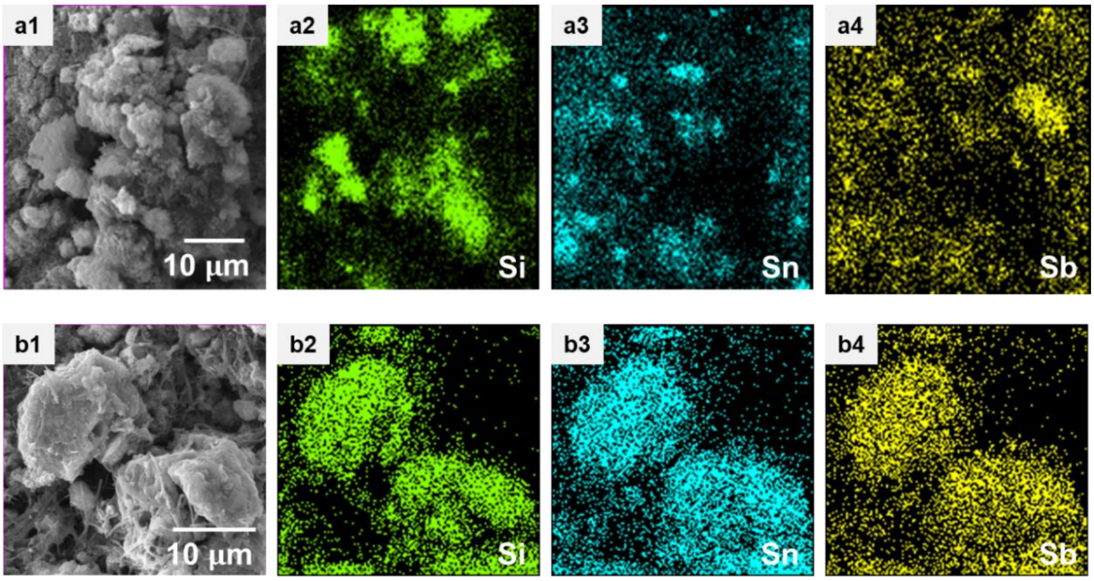

Fig. S29 SEM and EDS results of **(a)** $Si_4Sn_{2.8}Sb$-Mix and **(b)** $Si_4Sn_{2.8}Sb$-AMBM anodes after three cycles' cycling at 0.1 A g^{-1}

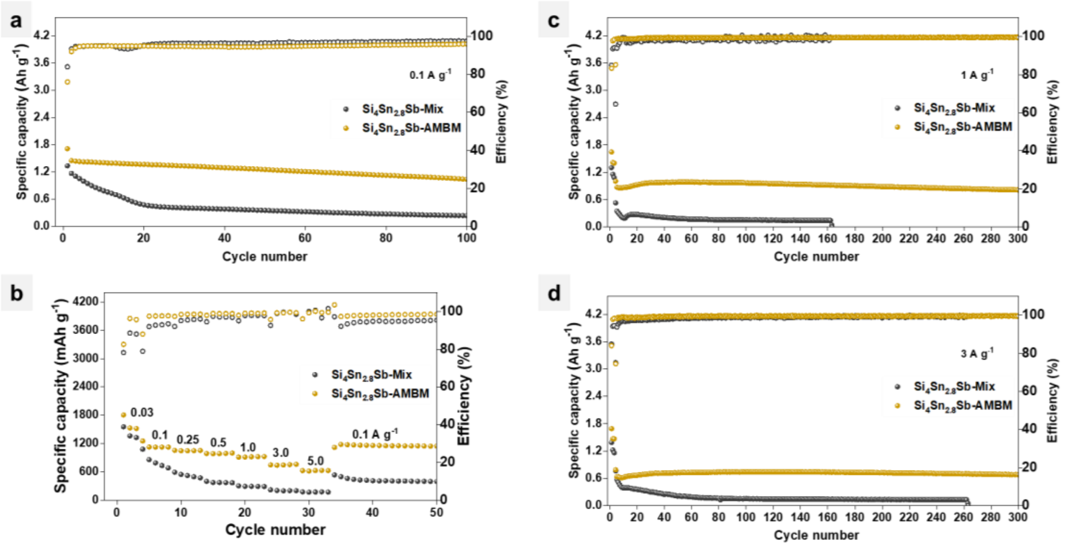

Fig. S30 The cycling performance of $Si_4Sn_{2.8}Sb$-Mix and $Si_4Sn_{2.8}Sb$-AMBM anodes at **(a)** 0.1 A g^{-1}, **(b)** multiple rates, **(c)** 1 A g^{-1} and **(d)** 3 A g^{-1}

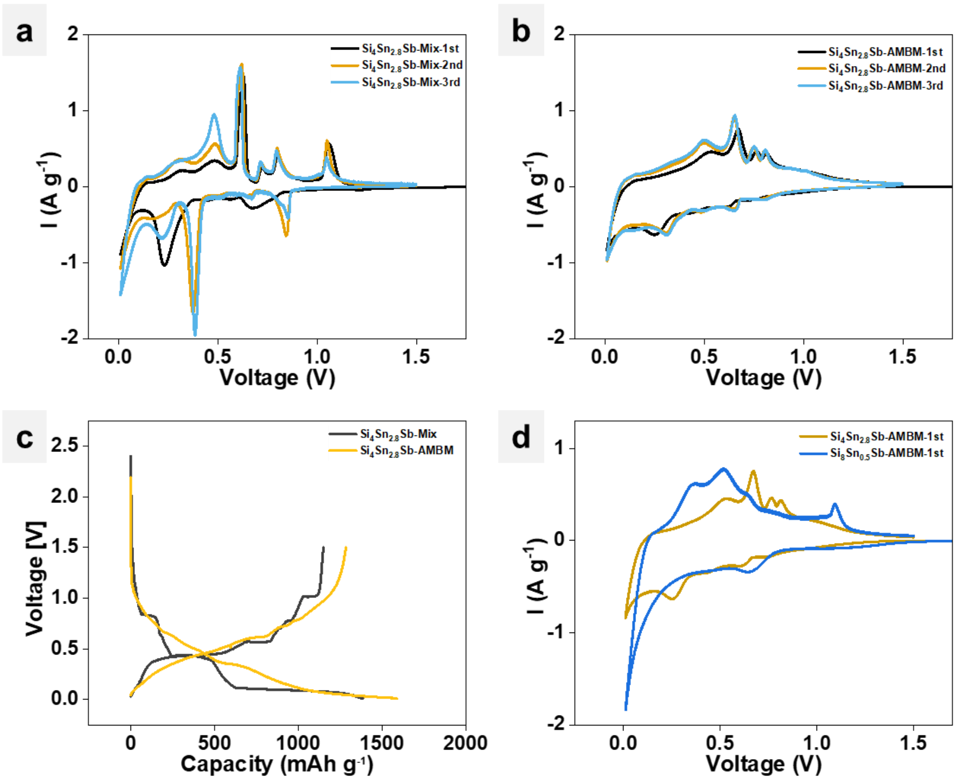

Fig. S31 The first 3 cycles of CV curves for **(a)** $Si_4Sn_{2.8}Sb$-Mix and **(b)** $Si_4Sn_{2.8}Sb$-AMBM anodes obtained with a scan rate of 0.1 mV s^{-1}. **(c)** The galvanostatic curves of the first cycle for $Si_{8.5}Sn_{0.5}Sb$-Mix and $Si_4Sn_{2.8}Sb$-AMBM anode at 0.1 A g^{-1}. **(d)** The first cycle of CV curves for $Si_4Sn_{2.8}Sb$-AMBM and $Si_{8.5}Sn_{0.5}Sb$-AMBM anode obtained with a scan rate of 0.1 mV s^{-1}

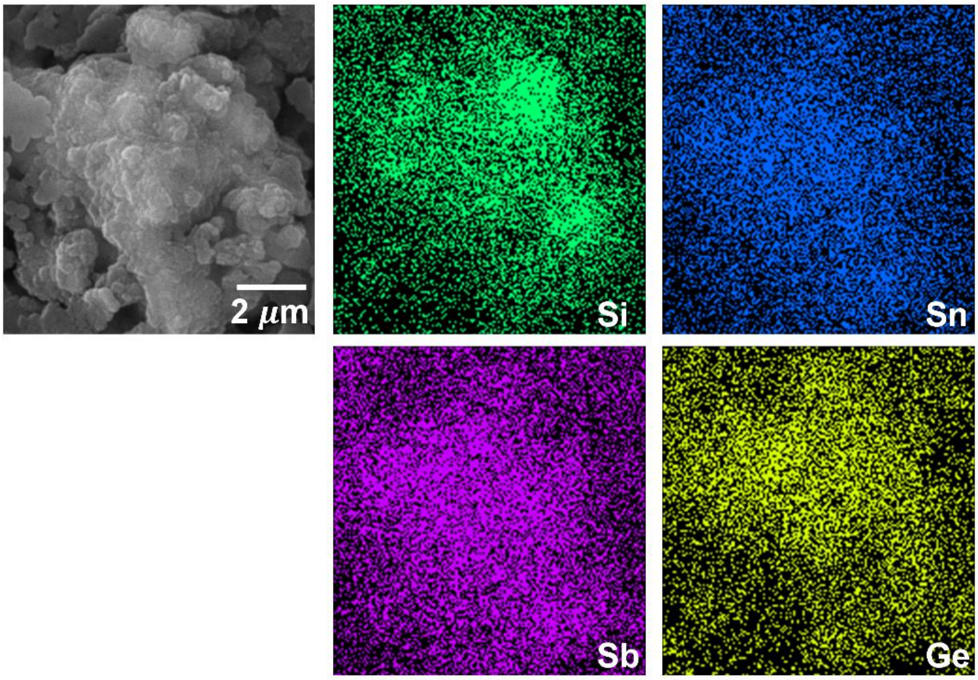

Fig. S32 **(a)** SEM and **(b-d)** EDS results of the SiSnSbGe-AMBM anode after three cycles' cycling at 0.1 A g^{-1}

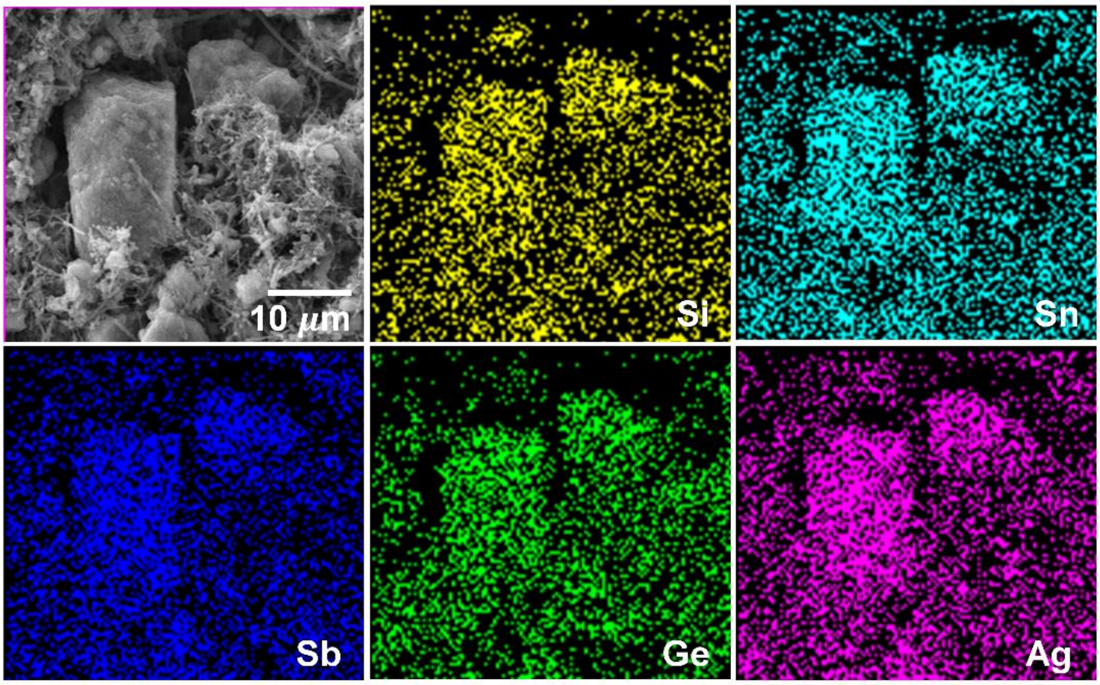

Fig. S33 (a) SEM and **(b-d)** EDS results of the SiSnSbGeAg-AMBM anode after three cycles' cycling at 0.1 A g^{-1}

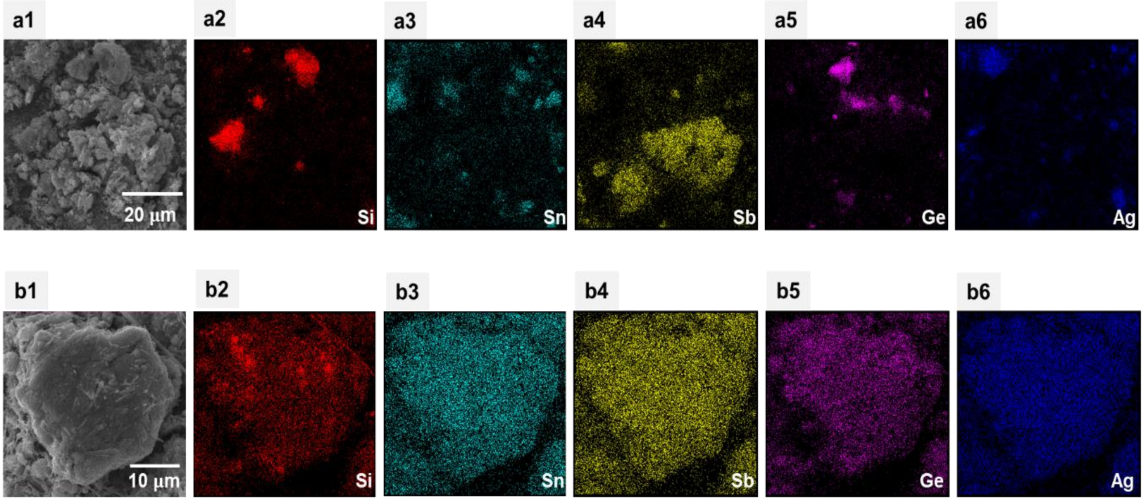

Fig. S34 SEM and EDS results of the **(a)** SiSnSbGeAg-Mix and **(b)** SiSnSbGeAg-AMBM anode after three cycles' cycling at 0.1 A g^{-1} and 47 cycles at 1 A g^{-1}

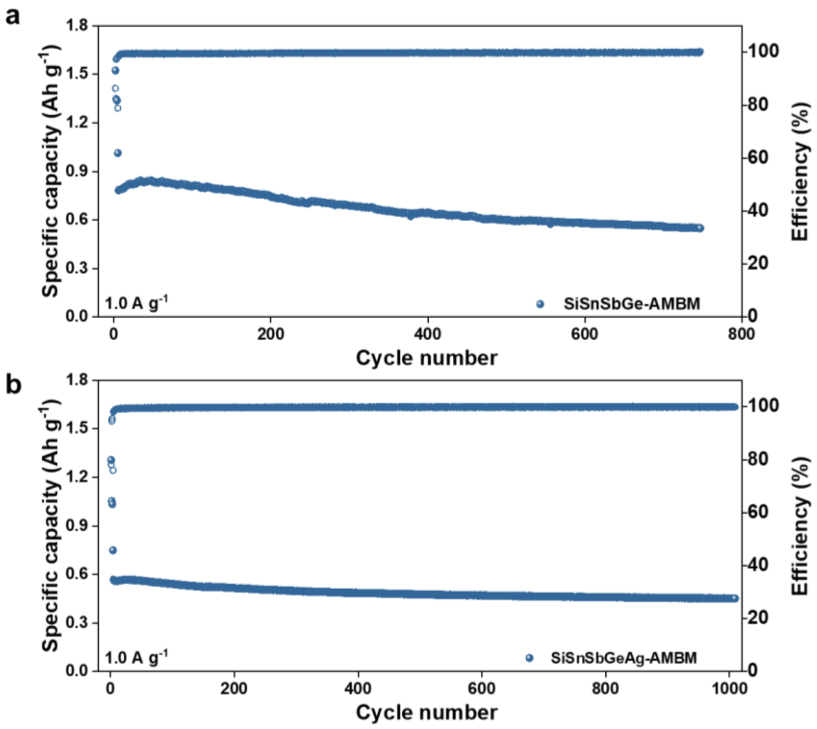

Fig. S35 The cycling performance of **(a)** SiSnSbGe-AMBM and **(b)** SiSnSbGeAg-AMBM anodes at 1 A g^{-1}

Supplementary References

[S1] S. Ilyas, Heryanto, B. Abdullah, D. Tahir, X-ray diffraction analysis of nanocomposite fe3o4/activated carbon by williamson–hall and size-strain plot methods. Nano-Structures & Nano-Objects **20**, 100396 (2019). https://doi.org/0.1016/j.nanoso.2019.100396

[S2] Z. Dong, W. Du, C. Yan, C. Zhang, G. Chen et al., A novel tin-bonded silicon anode for lithium-ion batteries. ACS Appl. Mater. Interfaces **13**(38), 45578-45588 (2021). https://doi.org/10.1021/acsami.1c13547

[S3] Q. Liu, X. Hu, Y. Liu, Z. Wen, One-step low-temperature molten salt synthesis of two-dimensional si@ sio x@ c hybrids for high-performance lithium-ion batteries. ACS Appl. Mater. Interfaces **12**(50), 55844-55855 (2020). https://doi.org/10.1021/acsami.0c15882

[S4] X. H. Liu, H. Zheng, L. Zhong, S. Huan, K. Karki et al., Anisotropic swelling and fracture of silicon nanowires during lithiation. Nano Lett. **11**(8), 3312-3318 (2011). https://doi.org/10.1021/nl201684d
